# The endocannabinoid gene *faah2a* modulates stress-associated behavior in zebrafish

**DOI:** 10.1371/journal.pone.0190897

**Published:** 2018-01-05

**Authors:** Randall G. Krug, Han B. Lee, Louis Y. El Khoury, Ashley N. Sigafoos, Morgan O. Petersen, Karl J. Clark

**Affiliations:** 1 Department of Biochemistry and Molecular Biology, Mayo Clinic, Rochester, MN, United States of America; 2 Mayo Clinic Graduate School of Biomedical Sciences (Neurobiology of Disease Track), Mayo Clinic, Rochester, MN, United States of America; 3 Mayo Clinic School of Medicine, Mayo Clinic, Rochester, MN, United States of America; Queen Mary University of London, UNITED KINGDOM

## Abstract

The ability to orchestrate appropriate physiological and behavioral responses to stress is important for survival, and is often dysfunctional in neuropsychiatric disorders that account for leading causes of global disability burden. Numerous studies have shown that the endocannabinoid neurotransmitter system is able to regulate stress responses and could serve as a therapeutic target for the management of these disorders. We used quantitative reverse transcriptase-polymerase chain reactions to show that genes encoding enzymes that synthesize (*abhd4*, *gde1*, *napepld)*, enzymes that degrade (*faah*, *faah2a*, *faah2b*), and receptors that bind (*cnr1*, *cnr2*, *gpr55-like*) endocannabinoids are expressed in zebrafish (*Danio rerio*). These genes are conserved in many other vertebrates, including humans, but fatty acid amide hydrolase 2 has been lost in mice and rats. We engineered transcription activator-like effector nucleases to create zebrafish with mutations in *cnr1* and *faah2a* to test the role of these genes in modulating stress-associated behavior. We showed that disruption of *cnr1* potentiated locomotor responses to hyperosmotic stress. The increased response to stress was consistent with rodent literature and served to validate the use of zebrafish in this field. Moreover, we showed for the first time that disruption of *faah2a* attenuated the locomotor responses to hyperosmotic stress. This later finding suggests that FAAH2 may be an important mediator of stress responses in non-rodent vertebrates. Accordingly, FAAH and FAAH2 modulators could provide distinct therapeutic options for stress-aggravated disorders.

## Introduction

Neuropsychiatric disorders are responsible for a devastating socioeconomic burden. They are a leading cause of disability and have afflicted approximately 1 in 5 adults during the past year [1,2]. Stress is an established risk factor for the onset and progression of these disorders, and thus there is a strong interest in identifying modulators of stress responses for therapeutic applications [3–6]. In recent years, the endocannabinoid (eCB) system has emerged as a candidate for these applications [7]. The lipid-derived neurotransmitters in this system are metabolized through multiple convergent and divergent biochemical pathways, and are able to signal through an array of cognate receptors [8]. The multipartite nature of this system provides an abundance of potential clinical targets that could be manipulated for the management of stress-aggravated disorders. For that reason a considerable amount of research has focused on clarifying how components of the eCB system regulate physiological and behavioral responses to stress.

The gene that has been most extensively investigated in this context encodes cannabinoid receptor 1, which is best known for its role in mediating the psychoactive affects of marijuana consumption [9]. However, the exogenous compounds that directly manipulate cannabinoid receptor 1 signaling have had limited clinical impact because of their adverse side effects [10]. Accordingly, many recent studies have concentrated on altering the activity of enzymes that metabolize endogenous cannabinoid receptor 1 ligands. Fatty acid amide hydrolase is an eCB catabolic enzyme that has been of particular interest because rodent models with genetic disruptions in *Faah* have increased eCB levels and decreased anxiety-like behaviors [11,12]. Numerous fatty acid amide hydrolase inhibitors have been developed, and *in vivo* testing has revealed their ability to similarly increase eCB levels and decrease stress-associated behavioral responses [13–15]. Nonetheless, the translational potential of these compounds are obscured to a certain extent because rodent models only have one gene encoding a fatty acid amide hydrolase while many non-rodent vertebrates, including humans, also have a *FAAH2* gene [16]. A recent case study has suggested that *FAAH2* may modulate anxiety in humans, and warranted the use of new model organisms to study the functions of fatty acid amide hydrolase homologues [17].

Zebrafish are a vertebrate model with a suite of characteristics that make them ideal for facilitating studies in this field of eCB biology, including a highly conserved eCB system with homologues of *FAAH2* [18–20]. Several groups have demonstrated that cannabinoid signaling modulates stress-associated behavior in adult zebrafish and a recent study confirmed the presence of the fatty acid amide hydrolase substrate anandamide (AEA) in developing zebrafish [21–25]. After considering these studies, we hypothesized that eCB signaling would modulate stress-associated behavior in larval zebrafish. To test this hypothesis, we first characterized the temporal expression patterns of eCB genes implicated in AEA signaling to determine which were expressed around the 5 dpf time point that coincides with neuroendocrine stress responses in larval zebrafish [26–28]. We subsequently used transcription activator-like effector nucleases (TALENs) to create zebrafish lines with indels in two eCB genes of interest, and examined how these disruptions affected locomotor responses to hyperosmotic stress. We studied *cnr1* because the existing rodent literature provided a valuable reference point for interpreting the results of our approach, and *faah2a* because the absence of studies on the *in vivo* functions of this gene magnified the potential clinical significance of our findings.

## Materials and methods

### Zebrafish husbandry

All zebrafish (*Danio rerio*) were maintained in accordance with protocols approved by the Institutional Animal Care and Use Committee at Mayo Clinic. Adult zebrafish lines were housed within the Mayo Clinic Zebrafish Core Facility, and mated in false bottom containers to generate offspring for line propagation and experimental purposes. Embryos obtained from individual pair crosses were mixed at 0 dpf for each experiment. The mixes were then divided into groups of 60 fish, which were transferred to 100 × 15 mm petri dishes (Becton, Dickinson and Company) containing 25 ml of 0.5X E2 media [29]. At 1 dpf, all nonviable embryos were removed from each group and the viable embryos were transferred to dishes containing 25 ml of fresh 0.5X E2 media. All embryos were raised in an incubator at 28.5°C with a 14/10-hour light/dark cycle until they were used in the experiments detailed in following Materials and methods subsections.

### Temporal patterns of eCB gene expression

Zebrafish were obtained from crosses between wild type fish, and raised according to the Materials and methods, Zebrafish husbandry subsection. The samples were collected at 0.25 dpf, 1.0 dpf, 2.0 dpf, 3.0 dpf, 4.0 dpf, 5.0 dpf, 6.0 dpf, and 7.0 dpf, and were treated and stored as previously described [28]. The temporal patterns of eCB gene expression were investigated with quantitative reverse transcriptase-polymerase chain reactions (qRT-PCRs). The samples were processed, ribonucleic acids (RNA) were isolated, complementary deoxyribonucleic acids were synthesized, and qRT-PCRs were performed as previously described [28]. The primers used in the qRT-PCRs were designed to amplify a region of the following eCB genes: *cnr1*, *cnr2*, *loc793909*, *abhd4*, *gde1*, *napepld*, *faah*, *faah2a*, and *faah2b* (S1A Table). Additional primers were designed and used to amplify regions of four selected reference genes previously used in zebrafish gene expression studies: *rps6kb1b* (Forward 5'-AAATCTCTATGGCGCTCGGACACC-3', Reverse 5'-TGGACTCCTTACACAGCCCGAAATC-3'), *eef1a1l1* (Forward 5'-TACAAATGCGGTGGAATCGACAAG-3', Reverse 5'-TCGGCCTTCAGTTTGTCCAACAC-3'), *rpl13a* (Forward 5'-TCTGGAGGACTGTAAGAGGTATGC-3', Reverse 5'-AGACGCACAATCTTGAGAGCAG-3'), and *b2m* (Forward 5'-GCCTTCACCCCAGAGAAAGG-3', Reverse 5'-GCGGTTG GGATTTACATGTTG-3') [30–32]. All primers were obtained from Integrated DNA Technologies (Integrated DNA Technologies Inc, Coralville, IA, USA). The obtained data was used to calculate mean expression ± 95% confidence intervals (95% CI) relative to the 5 dpf time point for each target gene. Comparisons between time points were made using a one-way analysis of variance (ANOVA) followed by Sidak's multiple comparisons test. All statistical analyses were performed using Prism 6 software (GraphPad Software, San Diego, CA, USA). To visualize a cross-comparison of all genes’ fold changes in this paper, heatmaps (S4–S11 Figs) were generated using the ‘gplots’ package in R software v3.4.1 (https://www.r-project.org). Fold changes were calculated using the 2^ΔCt^ formula for each gene pair combination when ΔCt was 0 or a positive number. However, when the ΔCt resulted in a negative value the following formula was used: -2^ΔCt^.

### Spatial patterns of eCB gene expression

Zebrafish in the experimental groups were obtained from crosses between wild type fish, while the zebrafish in the control groups were obtained from outcrosses between heterozygous *casz1*^mn0001Gt/+^ and wild type fish (*casz1*^+/+^) [30]. All fish were raised as described in the Materials and methods, Zebrafish husbandry subsection. The samples were collected at 2 dpf and 4 dpf as previously described [33]. Digoxigenin-labeled probes for *mRFP* were developed from cDNA clones as previously described [30]. Similarly, primers were obtained from Integrated DNA Technologies and used to develop digoxigenin-labeled probes for *faah*, *faah2a*, and *faah2b* (S1B Table). The *in situ* hybridization (ISH) experiment was performed using a previously published protocol with the following modifications [33]. In the modified protocol, the 2 dpf samples were treated with proteinase K (Roche) for 20 min and the 4 dpf samples were treated for 40 min. The proteinase K reactions were stopped with 3X 5 min PBT (Bio-Rad Laboratories) washes. The samples were refixed with a 4% PFA solution for 20 min, and then gently shaken during 5X 5 min PBT washes on a rotator (Fischer Scientific). The prehybridization step was completed using a 5 min wash with a 50% hybridization mix / 50% PBT solution, followed by a 4h incubation at 65°C with the hybridization mix. The hybridization step was completed using an overnight incubation at 65°C with hybridization mixes containing probes (1 ng/μl) for each gene of interest. Stringency washes were performed at 65°C to gradually replace the hybridization mixes with SSC buffer containing 0.1% Tween 20 (Bio-Rad Laboratories), which in turn was progressively replaced at room temperature with maleic acid buffer (0.1 M maleic acid, 0.15 M sodium chloride, pH = 7.5) containing 0.1% Tween 20 (MABT) (Bio-Rad Laboratories). The nonspecific antibody binding sites were blocked with 2% Blocking Reagent (Roche) in MABT. The samples were incubated in anti-digoxigenin-AP fab fragments antibody (Roche) diluted at 1:5,000 with blocking buffer. The antibody solution was removed and the samples were washed using 8X 15 min washes with MABT at room temperature. The samples were then equilibrated and stained. A series of single focal plane brightfield images was acquired for each sample using Specimen in a Corrected Optical Rotational Enclosure imaging techniques [34]. The image series were taken on an Axioplan 2 microscope (Carl Zeiss Microscopy) equipped with a Powershot G6 camera (Canon). Each series of images was compiled into a single composite image with Helicon Focus software (Helicon Soft).

### TALEN-mediated mutagenesis of eCB genes

The National Center for Biotechnology Information (NCBI) *Danio rerio* Annotation Release 105 was used to identify the predicted splice variants associated with each eCB gene of interest [35]. The Basic Local Alignment Search Tool (BLAST) was used to identify sequences encoding amino acid residues in *faah2a* that were putative homologues of the fatty acid amide hydrolase 2 serine-serine-lysine catalytic triad [16,35]. The TALENs were designed to recognize sites conserved in all predicted splice variants by using the Mojo Hand Version 2 software available online at http://www.talendesign.org [36]. The *cnr1* TALEN binding sites were positioned around a 15 base pair spacer sequence containing a BstUI restriction enzyme site (S1A Fig). The *faah2a* TALEN binding sites were positioned around a 13 base pair spacer sequence containing a BsrI restriction enzyme site just upstream of the sequence predicted to encode a catalytic lysine (S1B Fig). Primers were designed to flank these sites so that the fish could be genotyped via restriction fragment polymorphism (RFLP) analyses (S1C Table) [37]. The TALEN vectors were created using the Golden Gate method with a pT3TS-GoldyTALEN destination vector [37,38]. The TALEN mRNAs were synthesized and microinjected as previously described [37]. The fish harboring mutant eCB alleles were outcrossed with a dominant leopard line (Cx41.8^+/tq270^) to eventually establish populations of heterozygous F2 mutants [39]. The indels were characterized by sequencing, and the sperm from the F2 populations was cryopreserved [37]. The *cnr1* mutant line was assigned the Zebrafish Information Network (ZFIN) designator cnr1^mn49^, and the *faah2a* mutant line was assigned the ZFIN designator faah2a^mn50^ [40].

### Behavior assays

The zebrafish were obtained from in crosses between the F2 heterozygous eCB mutant lines described in the Materials and methods, TALEN-mediated mutagenesis of eCB genes subsection. All viable fish were raised according to the Materials and methods, Zebrafish husbandry subsection. At 3 dpf the fish were individually transferred in 400 μl of fresh 0.5X E2 media to wells on 48-well tissue culture plates (Corning). All of the behavior assays were completed using previously published protocols with the following modifications [41,42]. The 48-well plates were transferred from the incubator to light-box apparatuses 30 min after the onset of the light cycle on 5 dpf. All experiments were performed at 28.5°C, and the light-box apparatuses were configured with custom acrylic templates designed to align two 48-well plates. The apparatuses housed one pair of plates during each assay, with one plate serving as a control plate and the other as an experimental plate. During the hyperosmotic stress assays fish were acclimated to the apparatus for 45 min before the experiment was initiated. The pre-treatment locomotor baseline activity of the fish was filmed for 15 min, and then the treatments were applied to each plate. The control plates were treated with 100 μl of E2 media and the experimental plates were treated with 100 μl of a 500 mM sodium chloride (Sigma-Aldrich) solution prepared in E2 media (+100 mM final sodium chloride concentration). The post-treatment locomotor activity of the fish was filmed for 31 min. During the nicotine assays fish were acclimated to the apparatus for 20 min before the experiment was initiated. The pre-treatment baseline locomotor activity of the fish was filmed for 5 min, and then the treatments were applied to each plate. The control plates were treated with 100 μl of E2 media and the experimental plates were treated with 100 μl of a 250 μM nicotine (Acros Organics) solution prepared in E2 media (+50 μM final nicotine concentration). The post-treatment locomotor responses of the fish were then filmed for 5 min. The locomotor activity of the fish was analyzed at 1 second intervals with MATLAB software (The MathWorks, Natick, MA, USA) to calculate the distances travelled by each fish. After the behavior assays were completed, the individual larval zebrafish were genotyped using the RFLP analysis described in the Materials and methods, TALEN-mediated mutagenesis of eCB genes subsection. The locomotor data was used to calculate means ± 95% CI. Comparisons between groups were made using a two-way analysis of variance (ANOVA) followed by Tukey's honest significant difference test. All statistical analyses were performed using R software. All graphs were generated using R software, and Illustrator CC software (Adobe, San Jose, CA, USA).

## Results

### eCB gene expression occurs early in development

**Fig 1:**
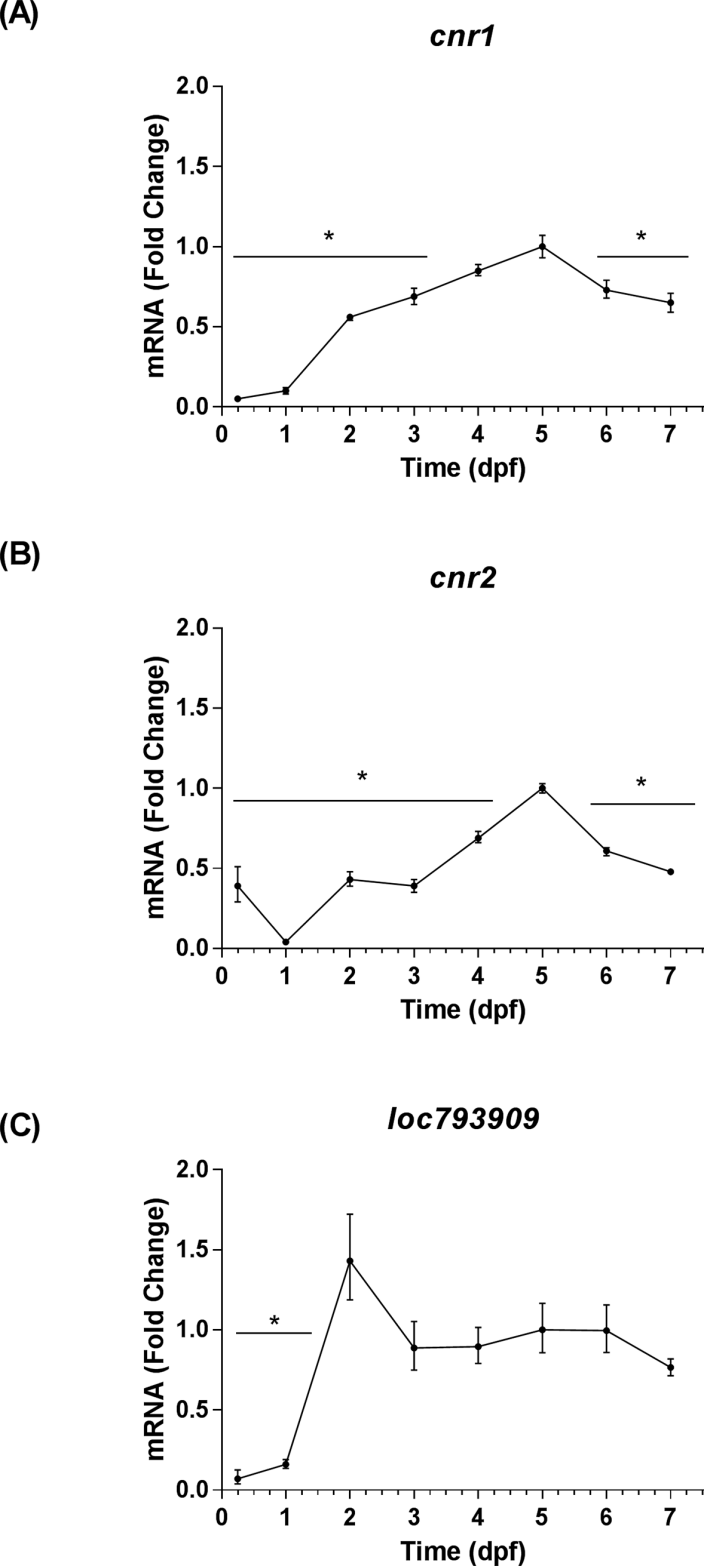
Temporal expression patterns of eCB receptor genes. The time points on all graphs are represented as means ± 95% CI (0.25–1 dpf: 20 larvae/n, n = 3; 2–7 dpf: 10 larvae/n, n = 3). * Indicates that a group is significantly different from the 5 dpf group (Sidak's multiple comparisons test, *P <* 0.05). (A) The fold change of *cnr1* transcript levels relative to 5 dpf as determined by qRT-PCR. (B) The fold change of *cnr2* transcript levels relative to 5 dpf as determined by qRT-PCR. (C) The fold change of *loc793909* transcript levels relative to 5 dpf as determined by qRT-PCR.

**Fig 2:**
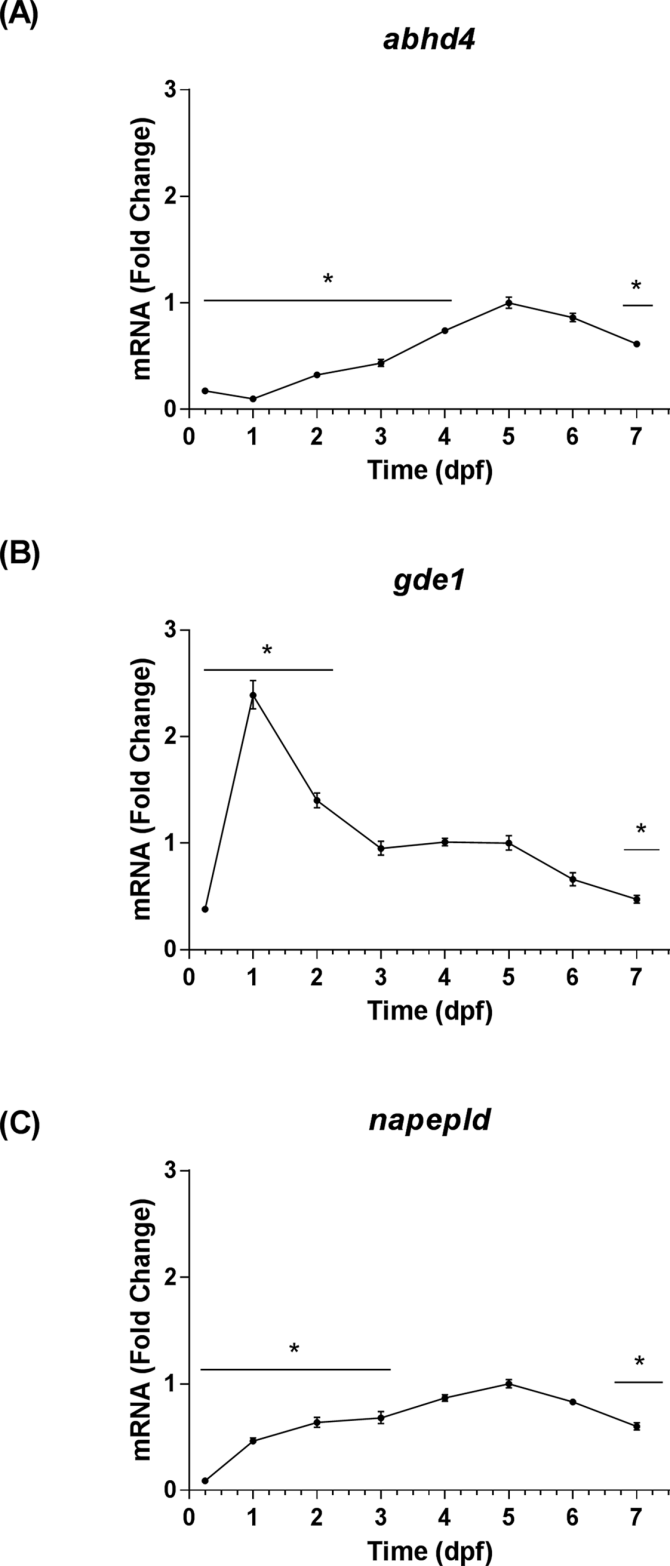
Temporal expression patterns of eCB anabolic enzyme genes. The time points on all graphs are represented as means ± 95% CI (0.25–1 dpf: 20 larvae/n, n = 3; 2–7 dpf: 10 larvae/n, n = 3). * Indicates that a group is significantly different from the 5 dpf group (Sidak's multiple comparisons test, *P <* 0.05). (A) The fold change of *abhd4* transcript levels relative to 5 dpf as determined by qRT-PCR. (B) The fold change of *gde1* transcript levels relative to 5 dpf as determined by qRT-PCR. (C) The fold change of *napepld* transcript levels relative to 5 dpf as determined by qRT-PCR.

**Fig 3:**
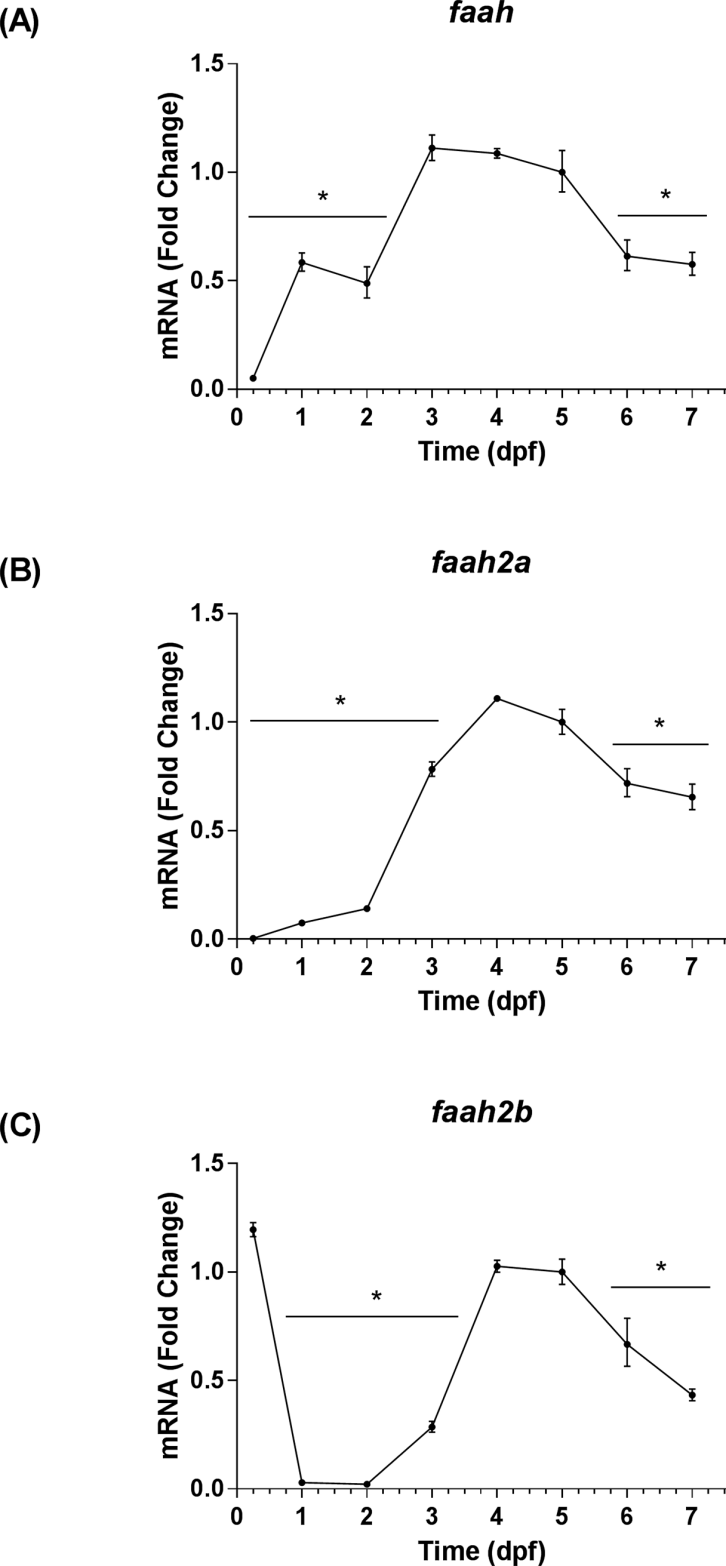
Temporal expression patterns of eCB catabolic enzyme genes. The time points on all graphs are represented as means ± 95% CI (0.25–1 dpf: 20 larvae/n, n = 3; 2–7 dpf: 10 larvae/n, n = 3). * Indicates that a group is significantly different from the 5 dpf group (Sidak's multiple comparisons test, *P <* 0.05). (A) The fold change of *faah* transcript levels relative to 5 dpf as determined by qRT-PCR. (B) The fold change of *faah2a* transcript levels relative to 5 dpf as determined by qRT-PCR. (C) The fold change of *faah2b* transcript levels relative to 5 dpf as determined by qRT-PCR.

The transcript levels of nine eCB genes were analyzed by qRT-PCR using *rps6kb1b* as the primary housekeeping gene. These levels were assessed relative to 5 dpf, because it corresponded with the stage of development used in our hyperosmotic stress assays. The stage of development significantly affected the expression levels of each eCB gene that was profiled [*cnr1*: *F*(7, 16) = 117.70, *P* < 0.0001; *cnr2*: *F*(7, 16) = 58.35, *P* < 0.0001; *loc793909*: *F*(7, 16) = 13.61, *P* < 0.0001; *abhd4*: *F*(7, 16) = 93.30, *P* < 0.0001; *gde1*: *F*(7, 16) = 58.94, *P* < 0.0001; *napepld*: *F*(7, 16) = 123.80, *P* < 0.0001; *faah*: *F*(7, 16) = 91.65, *P* < 0.0001; *faah2a*: *F*(7, 16) = 278.10, *P* < 0.0001; *faah2b*: *F*(7, 16) = 291.20, *P* < 0.0001]. The transcript levels of eCB receptors encoded by *cnr1* and *cnr2* increased to a peak at 5 dpf, and then exhibited declines at 6 dpf and 7 dpf (Fig 1A and 1B). The transcript levels of the eCB receptor encoded by *loc793909* increased at 2 dpf, and then exhibited an insignificant decrease that was followed by stability through 7 dpf (Fig 1C). The transcript levels of eCB anabolic enzymes encoded by *abhd4* and *napepld* increased through the first 5 dpf, and then exhibited declines at 6 dpf and 7 dpf (Fig 2A and 2C). In contrast, the transcript levels of the eCB anabolic enzyme encoded by *gde1* peaked at 1dpf, then declined through 7 dpf (Fig 2B). The transcript levels of eCB catabolic enzymes encoded by *faah* and *faah2a* increased through the first 4 dpf, then declined from 5–7 dpf (Fig 3A and 3B). The transcript levels of the eCB catabolic enzyme encoded by *faah2b* dropped between 0.25 dpf and 1dpf, subsequently increased through 4 dpf, and then declined again through 7 dpf (Fig 3C). The qRT-PCR threshold cycle (Ct) values recorded at 5 dpf for each eCB gene were included in the Supporting Information (S2A Table). The trends in eCB gene expression from 1–7 dpf were largely conserved regardless of the reference gene that was used, however, the stability of reference gene expression was not always conserved at the 0.25 dpf time point (S4–S11 Figs). In addition to the qRT-PCR experiments, ISH was used to investigate the spatiotemporal expression patterns of *faah*, *faah2a*, and *faah2b*. The expression of these genes were not detected by ISH in wild type fish at 2 dpf, however, they were detected at 4 dpf (S2A Fig). The expression of all three serine hydrolases was detected in the intestinal bulb. Additionally, faah and faah2a expression was detected in the liver. Background staining was accounted for by examining *mRFP* expression in a mix of siblings obtained by outcrossing heterozygous *casz1*^mn0001Gt/+^ fish with wild type (*casz1*^+/+^) fish [30]. The *mRFP* expression pattern of the GBT0001 line mimics that of *casz1*, and is only present in fish that harbor the gene-break transposon [30]. In these control fish—which were prepared alongside the experimental groups—no background staining was observed in the negative control wild type siblings while staining was observed in the positive control heterozygous *casz1*^mn0001Gt/+^ siblings (S2B Fig).

### eCB genes modulate stress-associated behavior

**Fig 4:**
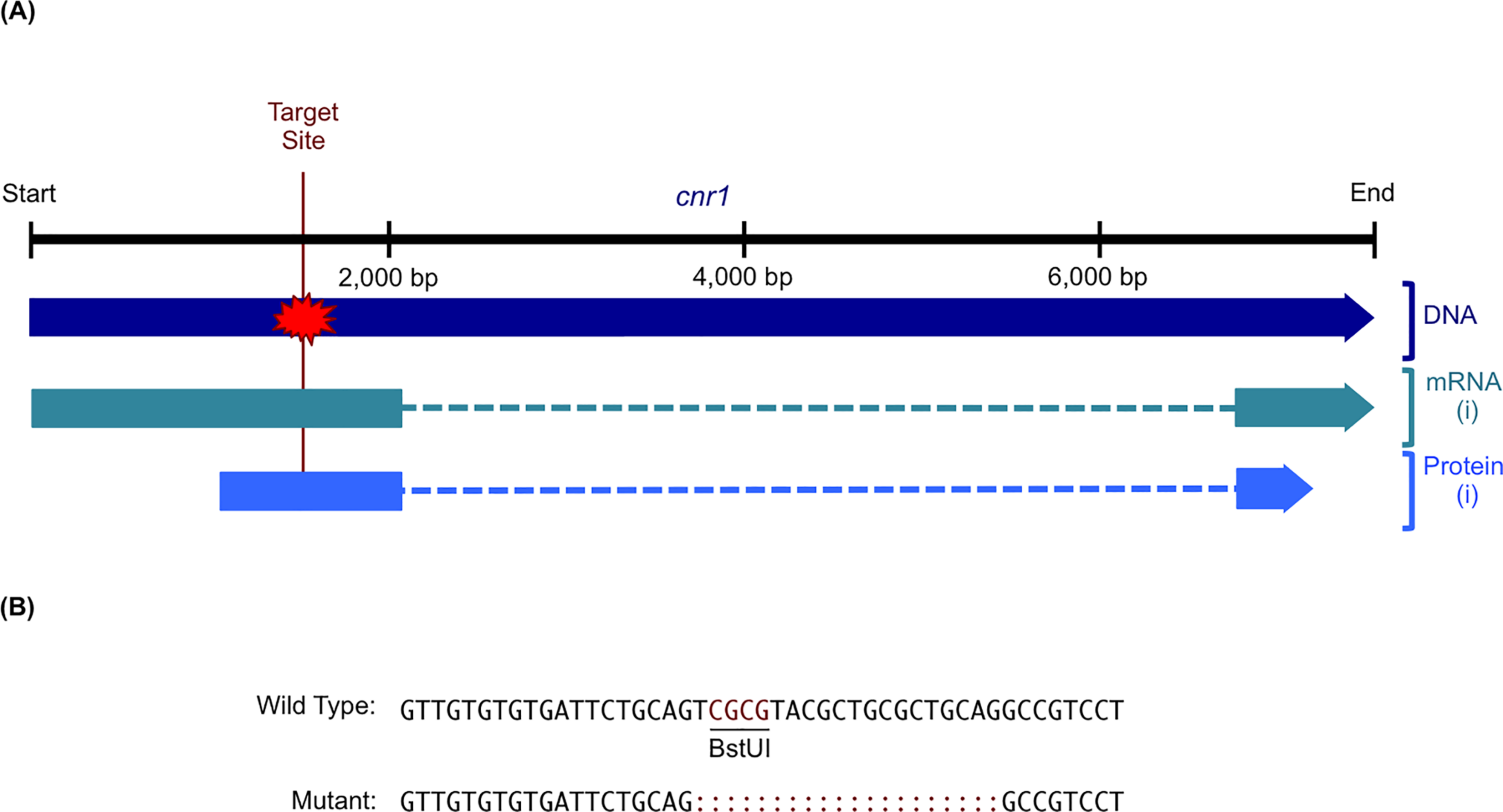
TALEN-mediated mutagenesis of *cnr1*. (A) The *cnr1* TALEN target site was designed in exon 1 so that mutagenesis would disrupt all predicted splice variants. NCBI Accessions: Gene ID, 404209; DNA, NC_007131.6; mRNA (i), NM_212820.1; Protein (i), NP_997985.1. (B) An alignment of wild type and mutant *cnr1* sequences reveals the TALEN-induced indel in the target BstUI restriction enzyme site.

**Fig 5:**
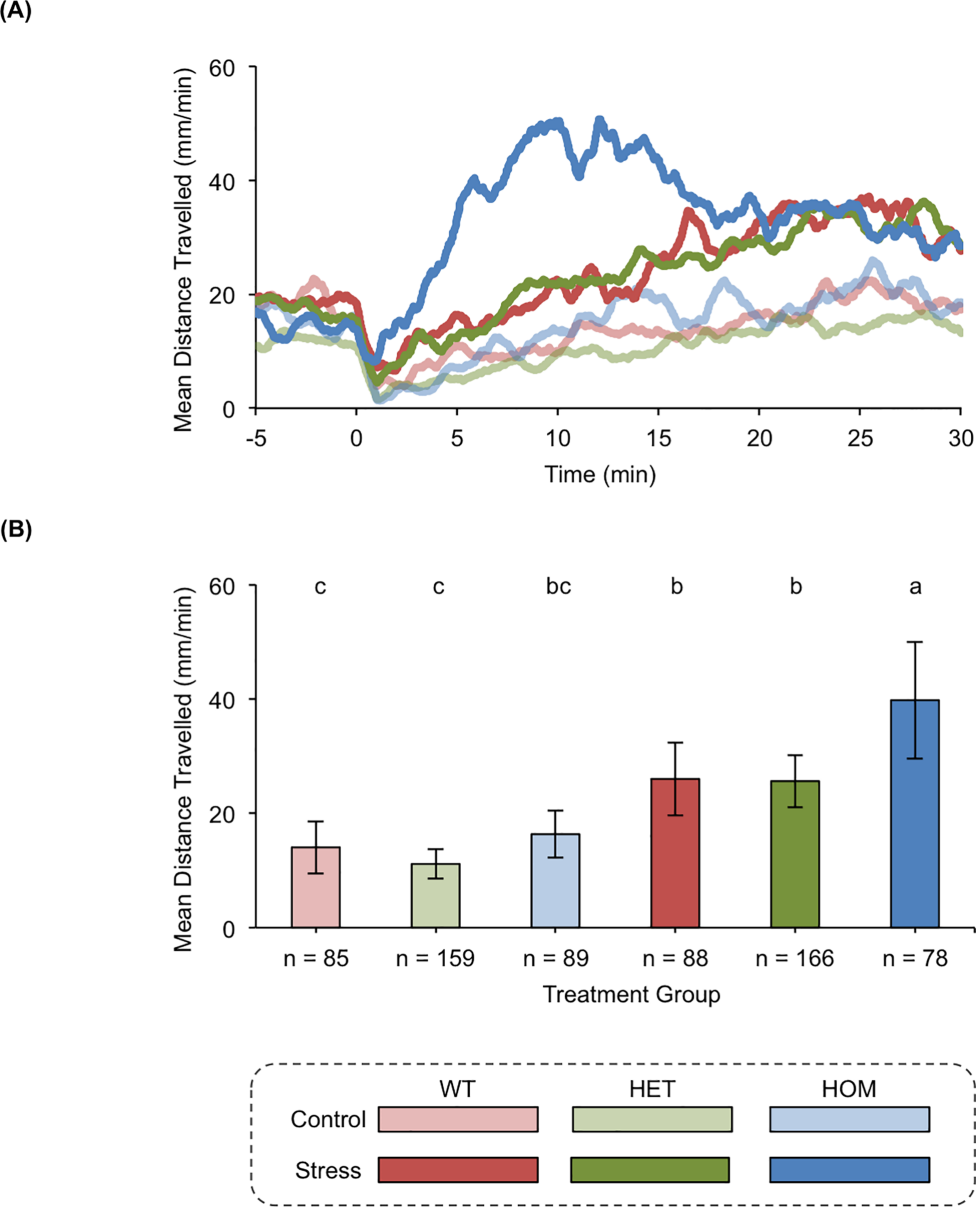
*cnr1* modulates stress-associated behavior. (A) The rolling means of distances travelled by wild type (WT), heterozygous *cnr1* (HET) mutant, and homozygous *cnr1* (HOM) mutant zebrafish. The pre-treatment baseline locomotor activity was recorded from −15–0 min, and the post-treatment locomotor activity was recorded from 0–31 min. At time 0 the zebrafish were treated with either E2 media (Control) or E2 media + NaCl (Stress). The locomotor activity at each second is represented as a mean of the distance travelled during the preceding 60 s. (B) The means of distances travelled after the zebrafish were treated with E2 media (Control) or E2 media + NaCl (Stress). The locomotor activity of each group is represented as a mean of the distance travelled per min during the 5–25 min time bin ± 95% CI. Groups with all different letters above the columns are statistically different from each other, while groups with a conserved letter above the columns are not statistically different from each other (Tukey's honest significant difference test, *P <* 0.05).

**Fig 6:**
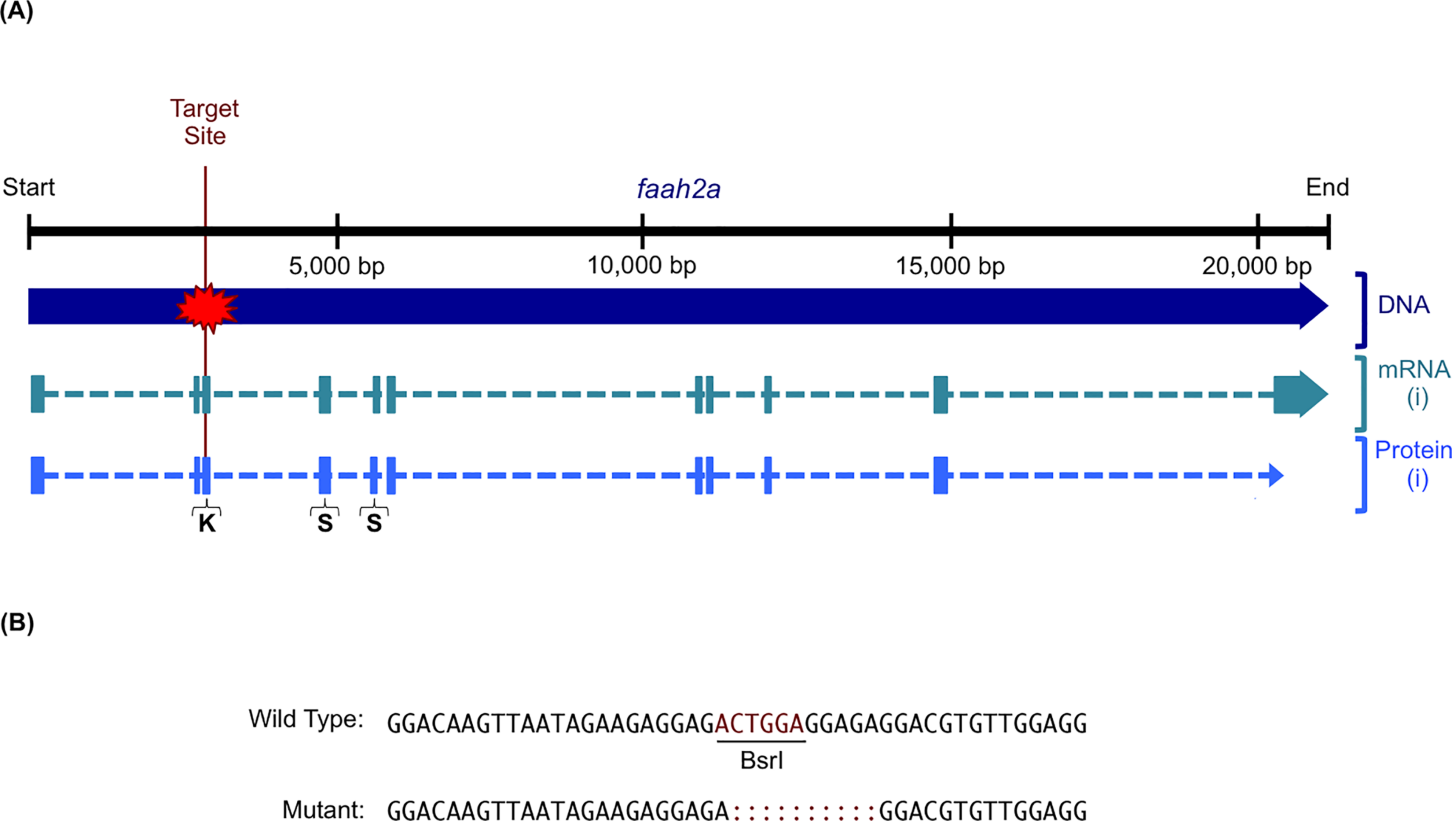
TALEN-mediated mutagenesis of *faah2a*. (A) The *faah2a* TALEN target site was designed in exon 3 so that mutagenesis would disrupt all predicted splice variants. NCBI Accessions: Gene ID, 436973; DNA, NC_007112.6; mRNA (i), NM_001002700.2; Protein (i), NP_001002700.1. The target site is just upstream of the sequence encoding a lysine in the predicted serine 228 (S) / serine 204 (S) / lysine 129 (K) catalytic triad. (B) An alignment of wild type and mutant *faah2a* sequences reveals the TALEN-induced indel in the target BsrI restriction enzyme site.

**Fig 7:**
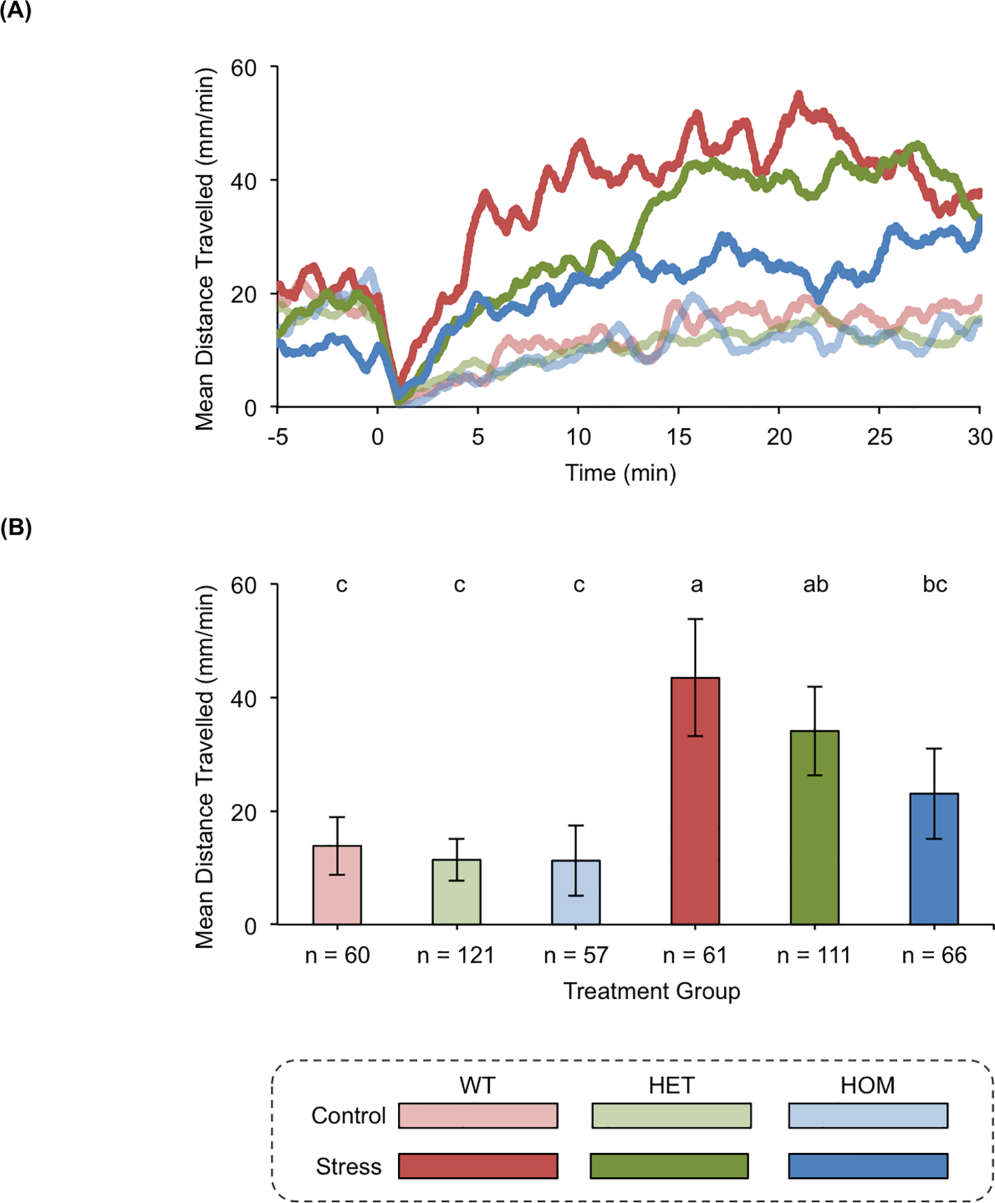
*faah2a* modulates stress-associated behavior. (A) The rolling means of distances travelled by wild type (WT), heterozygous *faah2a* (HET) mutant, and homozygous *faah2a* (HOM) mutant zebrafish. The pre-treatment baseline locomotor activity was recorded from −15–0 min, and the post-treatment locomotor activity was recorded from 0–31 min. At time 0 the zebrafish were treated with either E2 media (Control) or E2 media + NaCl (Stress). The locomotor activity at each second is represented as a mean of the distance travelled during the preceding 60 s. (B) The means of distances travelled after the zebrafish were treated with E2 media (Control) or E2 media + NaCl (Stress). The locomotor activity of each group is represented as a mean of the distance travelled per min during the 5–25 min time bin ± 95% CI. Groups with all different letters above the columns are statistically different from each other, while groups with a conserved letter above the columns are not statistically different from each other (Tukey's honest significant difference test, *P <* 0.05).

TALENs were used to generate zebrafish lines with frameshift mutations in the eCB receptor gene *cnr1* and the eCB metabolic enzyme gene *faah2a*. One pair of TALENs was designed for a target sequence in exon 1 of *cnr1* and used to generate a F2 heterozygous mutant line with a 20 base pair deletion (Fig 4, S1A Fig). This mutant line was then used to study how *cnr1* regulated stress-associated behavior (Fig 5A). Another pair of TALENs was designed for a target sequence in exon 3 of *faah2a* and used to generate a F2 heterozygous mutant line with a 10 base pair deletion (Fig 6, S1B Fig). Similarly, this mutant line was then used to study how *faah2a* regulated stress-associated behavior (Fig 7A). We have previously demonstrated that 5 dpf zebrafish elevate whole-body cortisol levels when challenged with hyperosmotic conditions, and that this neuroendocrine stress response correlated with an increase in stress-associated locomotion [28,42,43]. In the present study, a main affect of treatment was observed in the hyperosmotic stress assays performed with 5 dpf zebrafish obtained from F2 heterozygous eCB mutant in crosses [*cnr1*: *F*(1, 663) = 53.75, *P* < 0.0001; *faah2a*: *F*(1, 474) = 54.60, *P* < 0.0001]. A main affect of genotype was also observed in both of these assays [*cnr1*: *F*(2, 662) = 5.307, *P* < 0.01; *faah2a*: *F*(2, 473) = 3.502, *P* < 0.05]. No significant interaction was observed between treatment and genotype [*cnr1*: *F*(2, 659) = 2.114, *P* > 0.0.5; *faah2a*: *F*(2, 470) = 2.516, *P* > 0.05]. The wild type post-treatment Stress group exhibited a statistically significant increase in locomotor activity relative the wild type post-treatment Control group in each assay (Figs 5B and 7B). The homozygous *cnr1* mutant Stress group displayed a significantly higher locomotor response than the homozygous *cnr1* mutant Control group and the wild type Stress group (Fig 5B). The homozygous *faah2a* mutant stress group displayed a locomotor response that was not significantly different from the homozygous *faah2a* mutant Control group, and that was significantly lower than the wild type Stress group (Fig 7B). A nicotine assay was performed to demonstrate that the attenuated response of the homozygous *faah2a* mutants was specific to the hyperosmotic stress assay, and not simply attributed to a reduced capacity for locomotion (S3A Fig). Nicotine treatment has been shown to elicit robust locomotor responses from larval zebrafish, and in the present study a main affect of treatment was observed in the nicotine assay [*faah2a*: *F*(1, 90) = 81.51, *P* < 0.0001] [41,44]. No main affect of genotype was observed [*faah2a*: *F*(2, 89) = 1.890, *P* > 0.05], and no significant interaction between treatment and genotype was observed [*faah2a*: *F*(2, 86) = 1.224, *P* > 0.05]. There were no significant differences in the responses of each genotype to nicotine treatment (S3B Fig).

## Discussion

The eCB system is known to have a critical role in mediating responses to stress, including stress-associated behavior. For this reason, there is increasing interest in therapeutically manipulating the eCB system to manage a repertoire of stress-aggravated disorders. Even so, there is still a limited understanding of how individual eCB genes contribute to the regulation of stress responses. This interface could be rapidly explored by using a genetically amenable, high-throughput model organism like zebrafish. We started our study by assessing the ontogeny of zebrafish eCB gene expression in the first week of development to provide context for our later investigations of gene function. We specifically focused on genes that are implicated in AEA signaling because this eCB has been regarded as a gatekeeper of stress responses [45]. Most of the genes profiled in our qRT-PCR experiments, including *cnr1* and *faah2a*, exhibited time-dependent increases of corresponding mRNA levels. These data provided the most comprehensive analysis on the expression of these zebrafish genes to date and, when applicable, were generally consistent with previous reports [25,46–51]. We also used ISH to characterize the expression of serine hydrolases at 2 dpf and 4 dpf. Although no expression was detected at 2 dpf, expression was detected in the liver and intestinal bulb at 4 dpf. A previous qRT-PCR experiment detected *faah2a* in the brain of adult zebrafish, but we did not observe serine hydrolase expression in the nervous system at these stages of development [47]. It is possible that the whole-mount ISH protocol was not sensitive enough to detect low levels of gene expression that may exist there. A low level of eCB gene expression does not necessarily correlate with a lack of functional significance, and therefore we are reluctant to discount the potential physiological roles of these genes in the nervous system of larval zebrafish [52,53].

Nonetheless, the majority of the eCB genes we investigated had peak mRNA expression levels in the 4–5 dpf time bin, which corresponded with the onset of functioning physiological and behavioral stress responses [26–28,42,43,54]. Our subsequent hyperosmotic stress assay experiments confirmed that these genes had a role in modulating the stress-associated behavioral responses of larval zebrafish. When compared to wild type and heterozygous *cnr1* mutant siblings, the homozygous mutant siblings had a significantly higher locomotor response to hyperosmotic stress. The potentiated stress-associated behavioral response we observed in this zebrafish line was consistent with the behavior documented in rodent studies with cannabinoid receptor 1 mutant models [55]. These previous studies established that genetic disruption of cannabinoid receptor 1 caused anxiety-like behavior in numerous paradigms including elevated plus maze and light-dark tests [56–58]. Unlike cannabinoid receptor 1, little is known about fatty acid amide hydrolase 2 because this gene is not conserved in rodent models [16]. The single fatty acid amide hydrolase gene that is found in rodents, however, has been extensively researched. Stress-associated behaviors are reduced in rodent *Faah* mutant models, yet it is not clear how the function of this gene relates to the function of homologues found in organisms with multiple fatty acid amide hydrolase genes [11,12]. In our hyperosmotic stress assay, the homozygous *faah2a* zebrafish mutants exhibited significantly reduced locomotor responses to stress. These mutants did not have altered locomotor responses to nicotine treatment indicating that the attenuated response was specific to hyperosmotic stress. The reduced stress-associated behavioral response we observed in the *faah2a* mutants was similar to the behavior observed in rodent *Faah* mutants [11,12]. *In vitro* studies with human homologues of these genes have established that they both catabolize the eCB AEA, and so we suspect that disrupted AEA signaling could account for the observed phenotype because of its well-documented role in modulating responses to stress [16]. However, other less characterized eCBs like oleamide and ethanolamine variants are also known to be substrates of fatty acid amide hydrolases, and could contribute to the phenotype [16].

The experiments with our *cnr1* mutant line indicated that eCB signaling has a conserved role in modulating the stress-associated behavior of larval zebrafish, and that the unique advantages of this model organism could be leveraged to advance the field of eCB biology. Indeed, we developed the first animal model featuring a mutation in a homologue of *FAAH2* and provided evidence that this gene is involved with regulating stress responses. These results suggest that FAAH2 modulators could potentially be used as a new pharmacotherapeutic class of compounds for manipulating AEA signaling and managing stress-aggravated disorders. This potential should be further investigated by interrogating the functions of all three fatty acid amide hydrolase genes found in zebrafish, which include one *FAAH* homologue and two *FAAH2* paralogues [18–20]. The development of lines with a mutation in each of these genes would deliver a platform for beginning to clarify the redundancies and discrepancies that may exist in the functions of different fatty acid amide hydrolases. Distinguishing between the functions of *FAAH* and *FAAH2* would enable a more refined approach to any downstream preclinical applications. While the results of this study are an important step towards this end, we recognize the need to assess the role of these genes in additional behavioral paradigms. Larval zebrafish have been increasingly used to study responses to stress and a number of assays could be adapted for this purpose including edge preference or light/dark tests [43,59,60]. The changes in stress-associated behavior should also be assessed in adults because it could ensure that the phenotypes are not specific to the stage of development, and would enhance the translational potential of the results. Additionally, we believe it is important to develop strategies for correlating any changes observed in these tests with alterations in eCB signaling dynamics and physiological neuroendocrine stress responses. By making the mutant lines detailed in this paper readily available to the scientific community, we hope to facilitate these studies and to help unlock the eCB system's potential to improve human health.

## Supporting information

S1 Fig TALEN-mediated mutagenesis of eCB genes. (A) A schematic of the *cnr1* exon 1 TALEN target sequence and RFLP analysis region. L Binding Site, left TALEN binding site; R Binding Site, right TALEN binding site; F, forward primer; R, reverse primer; BstUI, BstUI restriction enzyme site (highlighted). (B) A schematic of the *faah2a* exon 3 TALEN target site and RFLP analysis region. L Binding Site, left TALEN binding site; R Binding Site, right TALEN binding site; F, forward primer; R, reverse primer; BsrI, BsrI rectriction enzyme site (highlighted).
(TIF)

S2 Fig Spatial expression patterns of serine hydrolase genes. (A) The expression patterns of *faah*, *faah2a*, and *faah2b* as determined by ISH. The expression patterns were assessed in samples of wild type (WT) zebrafish siblings that were collected at 2 dpf and 4 dpf. (B) The expression patterns of *mRFP* as determined by ISH. The expression patterns were assessed in samples of wild type (WT) and heterozygous *casz1*^mn0001Gt/+^zebrafish siblings that were collected at 2 dpf and 4 dpf.
(TIF)

S3 Fig faah2a does not modulate locomotor responses to nicotine treatment. (A) The rolling means of distances travelled by wild type (WT), heterozygous *faah2a* (HET) mutant, and homozygous *faah2a* (HOM) mutant zebrafish. The pre-treatment baseline locomotor activity was recorded from −5–0 min, and the post-treatment locomotor activity was recorded from 0–5 min. At time 0 the zebrafish were treated with either E2 media (Control) or E2 media + Nicotine (Nicotine). The locomotor activity at each second is represented as a mean of the distance travelled during the preceding 60 s. (B) The means of distances travelled after the zebrafish were treated with E2 media (Control) or E2 media + Nicotine (Nicotine). The locomotor activity of each group is represented as a mean of the distance travelled per min during the 0–4 min time bin ± 95% CI. Groups with all different letters above the columns are statistically different from each other, while groups with a conserved letter above the columns are not statistically different from each other (Tukey's honest significant difference test, *P <* 0.05).
(TIF)

S4 Fig Heatmaps showing the fold change of each gene pair combination through 7 dpf. Fold change is calculated using the 2^ΔCt^ formula when ΔCt is greater or equal to 0, and -2^ΔCt^ when ΔCt is less than 0 for each gene pair combination. The coloring of the heatmaps is based on the calculation of the ΔCt by subtracting the Ct of the gene in the row from the Ct of the gene in the column (ΔCt = Ct_column_−Ct_row_). S4 Fig: 0.25 dpf, S5 Fig: 1 dpf, S6 Fig: 2 dpf, S7 Fig: 3 dpf, S8 Fig: 4 dpf, S9 Fig: 5 dpf, S10 Fig: 6 dpf, S11 Fig: 7 dpf.
(TIF)

S5 Fig Heatmaps showing the fold change of each gene pair combination through 7 dpf. Fold change is calculated using the 2^ΔCt^ formula when ΔCt is greater or equal to 0, and -2^ΔCt^ when ΔCt is less than 0 for each gene pair combination. The coloring of the heatmaps is based on the calculation of the ΔCt by subtracting the Ct of the gene in the row from the Ct of the gene in the column (ΔCt = Ct_column_−Ct_row_). S4 Fig: 0.25 dpf, S5 Fig: 1 dpf, S6 Fig: 2 dpf, S7 Fig: 3 dpf, S8 Fig: 4 dpf, S9 Fig: 5 dpf, S10 Fig: 6 dpf, S11 Fig: 7 dpf.
(TIF)

S6 Fig Heatmaps showing the fold change of each gene pair combination through 7 dpf. Fold change is calculated using the 2^ΔCt^ formula when ΔCt is greater or equal to 0, and -2^ΔCt^ when ΔCt is less than 0 for each gene pair combination. The coloring of the heatmaps is based on the calculation of the ΔCt by subtracting the Ct of the gene in the row from the Ct of the gene in the column (ΔCt = Ct_column_−Ct_row_). S4 Fig: 0.25 dpf, S5 Fig: 1 dpf, S6 Fig: 2 dpf, S7 Fig: 3 dpf, S8 Fig: 4 dpf, S9 Fig: 5 dpf, S10 Fig: 6 dpf, S11 Fig: 7 dpf.
(TIF)

S7 Fig Heatmaps showing the fold change of each gene pair combination through 7 dpf. Fold change is calculated using the 2^ΔCt^ formula when ΔCt is greater or equal to 0, and -2^ΔCt^ when ΔCt is less than 0 for each gene pair combination. The coloring of the heatmaps is based on the calculation of the ΔCt by subtracting the Ct of the gene in the row from the Ct of the gene in the column (ΔCt = Ct_column_−Ct_row_). S4 Fig: 0.25 dpf, S5 Fig: 1 dpf, S6 Fig: 2 dpf, S7 Fig: 3 dpf, S8 Fig: 4 dpf, S9 Fig: 5 dpf, S10 Fig: 6 dpf, S11 Fig: 7 dpf.
(TIF)

S8 Fig Heatmaps showing the fold change of each gene pair combination through 7 dpf. Fold change is calculated using the 2^ΔCt^ formula when ΔCt is greater or equal to 0, and -2^ΔCt^ when ΔCt is less than 0 for each gene pair combination. The coloring of the heatmaps is based on the calculation of the ΔCt by subtracting the Ct of the gene in the row from the Ct of the gene in the column (ΔCt = Ct_column_−Ct_row_). S4 Fig: 0.25 dpf, S5 Fig: 1 dpf, S6 Fig: 2 dpf, S7 Fig: 3 dpf, S8 Fig: 4 dpf, S9 Fig: 5 dpf, S10 Fig: 6 dpf, S11 Fig: 7 dpf.
(TIF)

S9 Fig Heatmaps showing the fold change of each gene pair combination through 7 dpf. Fold change is calculated using the 2^ΔCt^ formula when ΔCt is greater or equal to 0, and -2^ΔCt^ when ΔCt is less than 0 for each gene pair combination. The coloring of the heatmaps is based on the calculation of the ΔCt by subtracting the Ct of the gene in the row from the Ct of the gene in the column (ΔCt = Ct_column_−Ct_row_). S4 Fig: 0.25 dpf, S5 Fig: 1 dpf, S6 Fig: 2 dpf, S7 Fig: 3 dpf, S8 Fig: 4 dpf, S9 Fig: 5 dpf, S10 Fig: 6 dpf, S11 Fig: 7 dpf.
(TIF)

S10 Fig Heatmaps showing the fold change of each gene pair combination through 7 dpf. Fold change is calculated using the 2^ΔCt^ formula when ΔCt is greater or equal to 0, and -2^ΔCt^ when ΔCt is less than 0 for each gene pair combination. The coloring of the heatmaps is based on the calculation of the ΔCt by subtracting the Ct of the gene in the row from the Ct of the gene in the column (ΔCt = Ct_column_−Ct_row_). S4 Fig: 0.25 dpf, S5 Fig: 1 dpf, S6 Fig: 2 dpf, S7 Fig: 3 dpf, S8 Fig: 4 dpf, S9 Fig: 5 dpf, S10 Fig: 6 dpf, S11 Fig: 7 dpf.
(TIF)

S11 Fig Heatmaps showing the fold change of each gene pair combination through 7 dpf. Fold change is calculated using the 2^ΔCt^ formula when ΔCt is greater or equal to 0, and -2^ΔCt^ when ΔCt is less than 0 for each gene pair combination. The coloring of the heatmaps is based on the calculation of the ΔCt by subtracting the Ct of the gene in the row from the Ct of the gene in the column (ΔCt = Ct_column_−Ct_row_). S4 Fig: 0.25 dpf, S5 Fig: 1 dpf, S6 Fig: 2 dpf, S7 Fig: 3 dpf, S8 Fig: 4 dpf, S9 Fig: 5 dpf, S10 Fig: 6 dpf, S11 Fig: 7 dpf.
(TIF)

S1 Table Primers for eCB gene analyses. (A) A list of select zebrafish eCB genes and the primers used to amplify target regions in them for qRT-PCR analyses. (B) A list of select zebrafish eCB genes and the primers used to amplify target regions in them for ISH analyses. (C) A list of select zebrafish eCB genes and the primers used to amplify target regions in them for RFLP analyses.
(TIF)

S2 Table qRT-PCR Ct values. (A) A list of Ct values for select zebrafish eCB and reference gene pairs at 5 dpf. The values are shown as a mean ± SD (10 larvae/n, n = 3).
(TIF)
