# Supplementary figures and images for: The endocannabinoid gene faah2a modulates stress-associated behavior in zebrafish
Source: PLoS One. 2018 Jan 5;13(1):e0190897. doi: 10.1371/journal.pone.0190897 (PMC5756047; doi:10.1371/journal.pone.0190897)

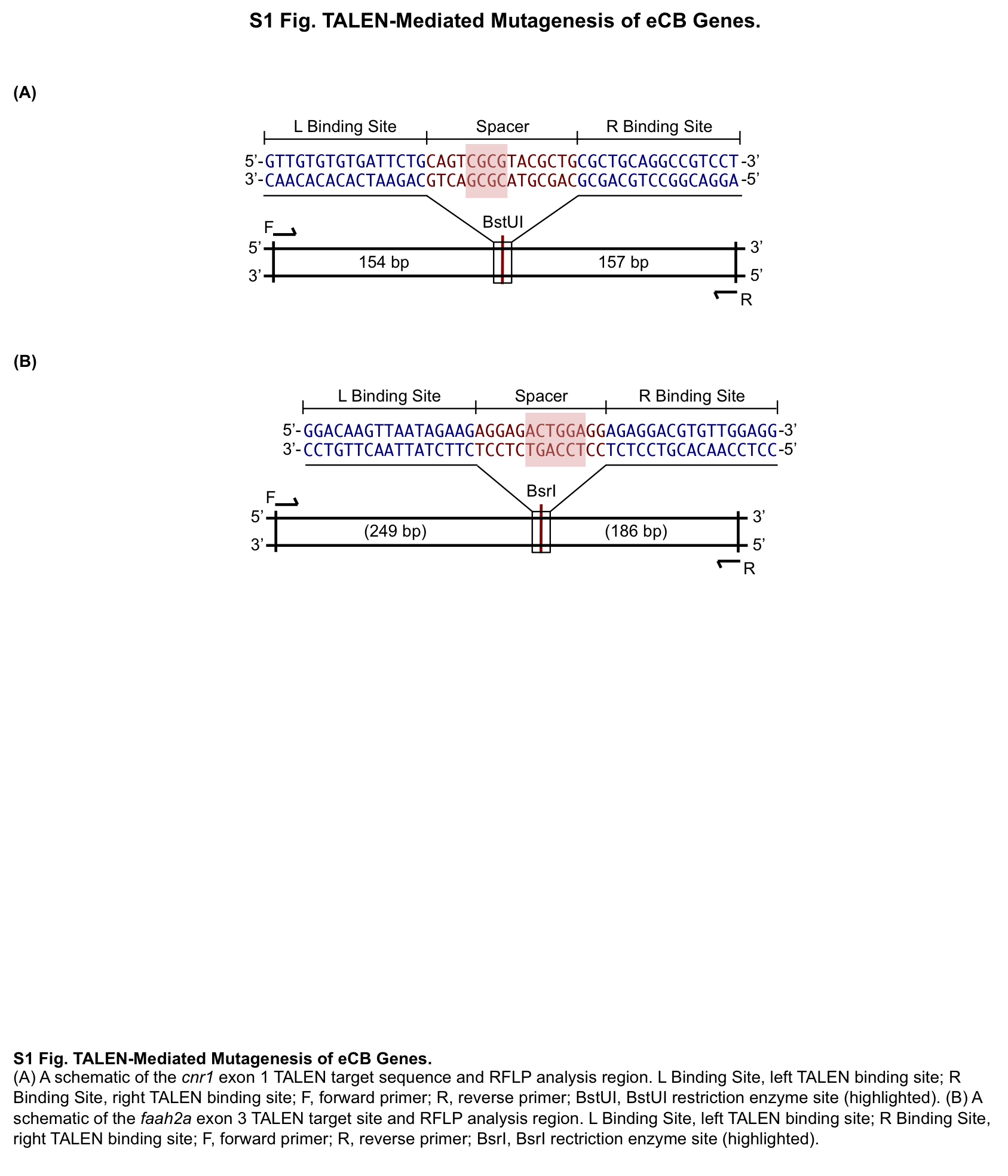

Supplement: S1 Fig — (A) A schematic of the cnr1 exon 1 TALEN target sequence and RFLP analysis region. L Binding Site, left TALEN binding site; R Binding Site, right TALEN binding site; F, forward primer; R, reverse primer; BstUI, BstUI restriction enzyme site (highlighted). (B) A schematic of the faah2a exon 3 TALEN target site and RFLP analysis region. L Binding Site, left TALEN binding site; R Binding Site, right TALEN binding site; F, forward primer; R, reverse primer; BsrI, BsrI rectriction enzyme site (highlighted). (TIF) [file pone.0190897.s001.tif]

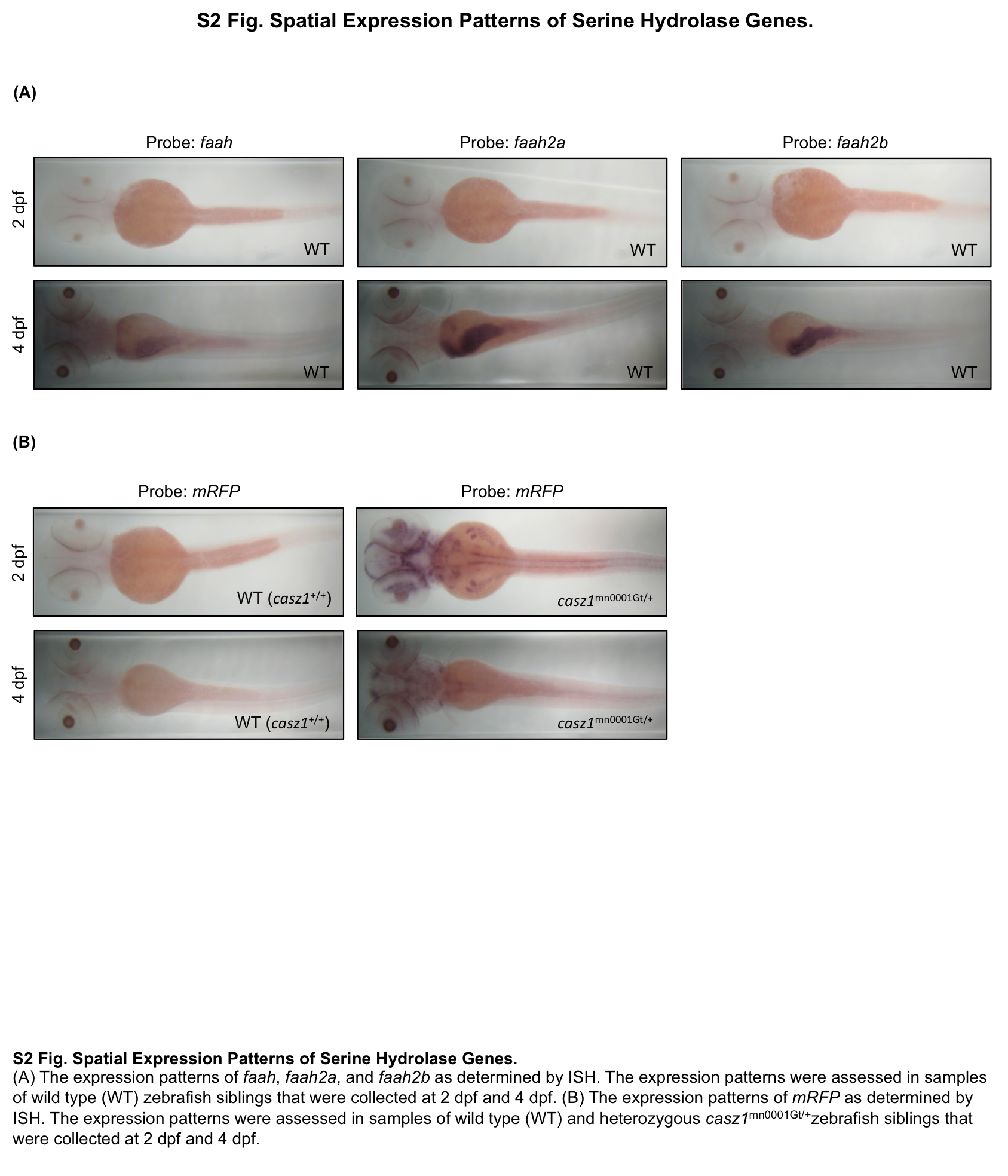

Supplement: S2 Fig — (A) The expression patterns of faah, faah2a, and faah2b as determined by ISH. The expression patterns were assessed in samples of wild type (WT) zebrafish siblings that were collected at 2 dpf and 4 dpf. (B) The expression patterns of mRFP as determined by ISH. The expression patterns were assessed in samples of wild type (WT) and heterozygous casz1mn0001Gt/+zebrafish siblings that were collected at 2 dpf and 4 dpf. (TIF) [file pone.0190897.s002.tif]

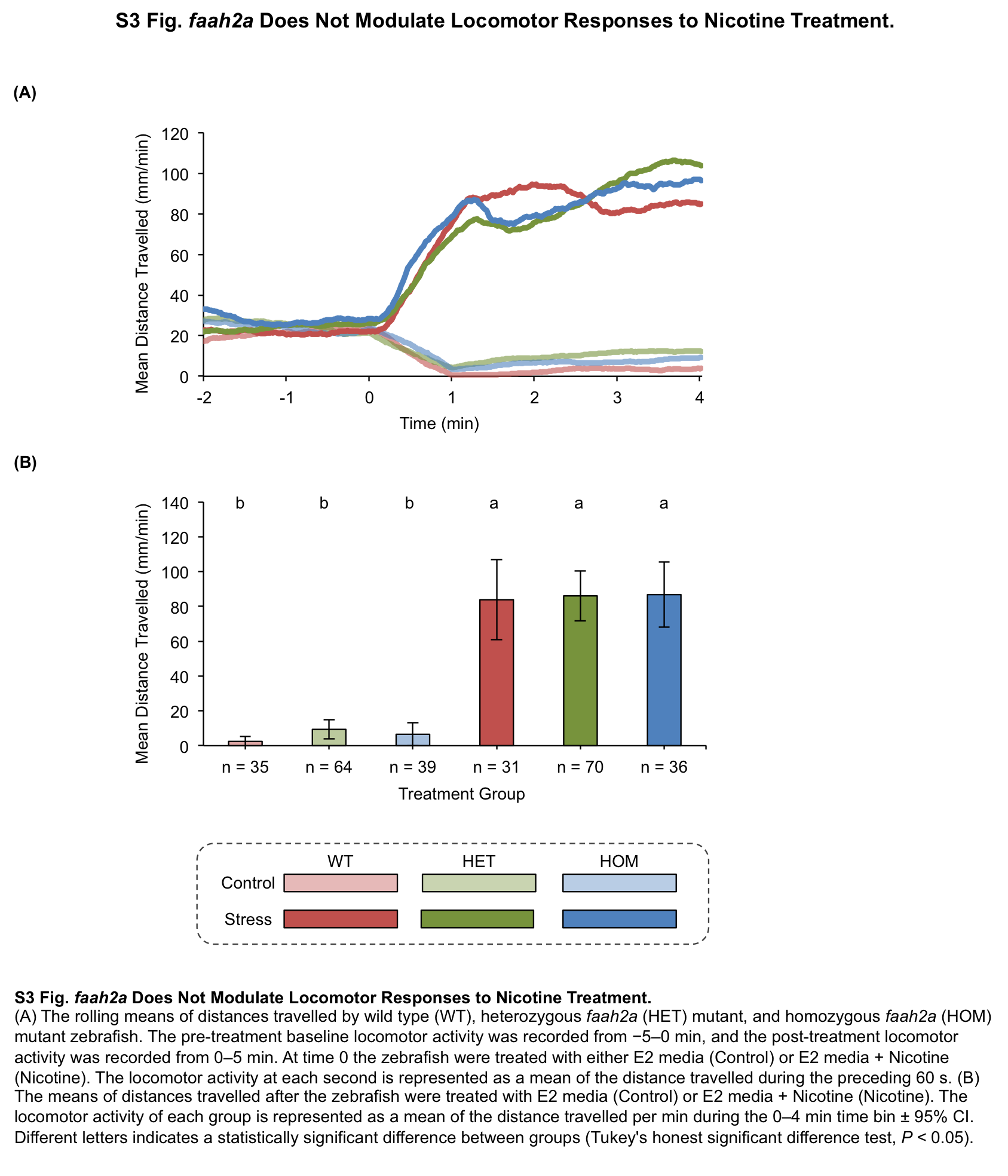

Supplement: S3 Fig — (A) The rolling means of distances travelled by wild type (WT), heterozygous faah2a (HET) mutant, and homozygous faah2a (HOM) mutant zebrafish. The pre-treatment baseline locomotor activity was recorded from −5–0 min, and the post-treatment locomotor activity was recorded from 0–5 min. At time 0 the zebrafish were treated with either E2 media (Control) or E2 media + Nicotine (Nicotine). The locomotor activity at each second is represented as a mean of the distance travelled during the preceding 60 s. (B) The means of distances travelled after the zebrafish were treated with E2 media (Control) or E2 media + Nicotine (Nicotine). The locomotor activity of each group is represented as a mean of the distance travelled per min during the 0–4 min time bin ± 95% CI. Groups with all different letters above the columns are statistically different from each other, while groups with a conserved letter above the columns are not statistically different from each other (Tukey's honest significant difference test, P < 0.05). (TIF) [file pone.0190897.s003.tif]

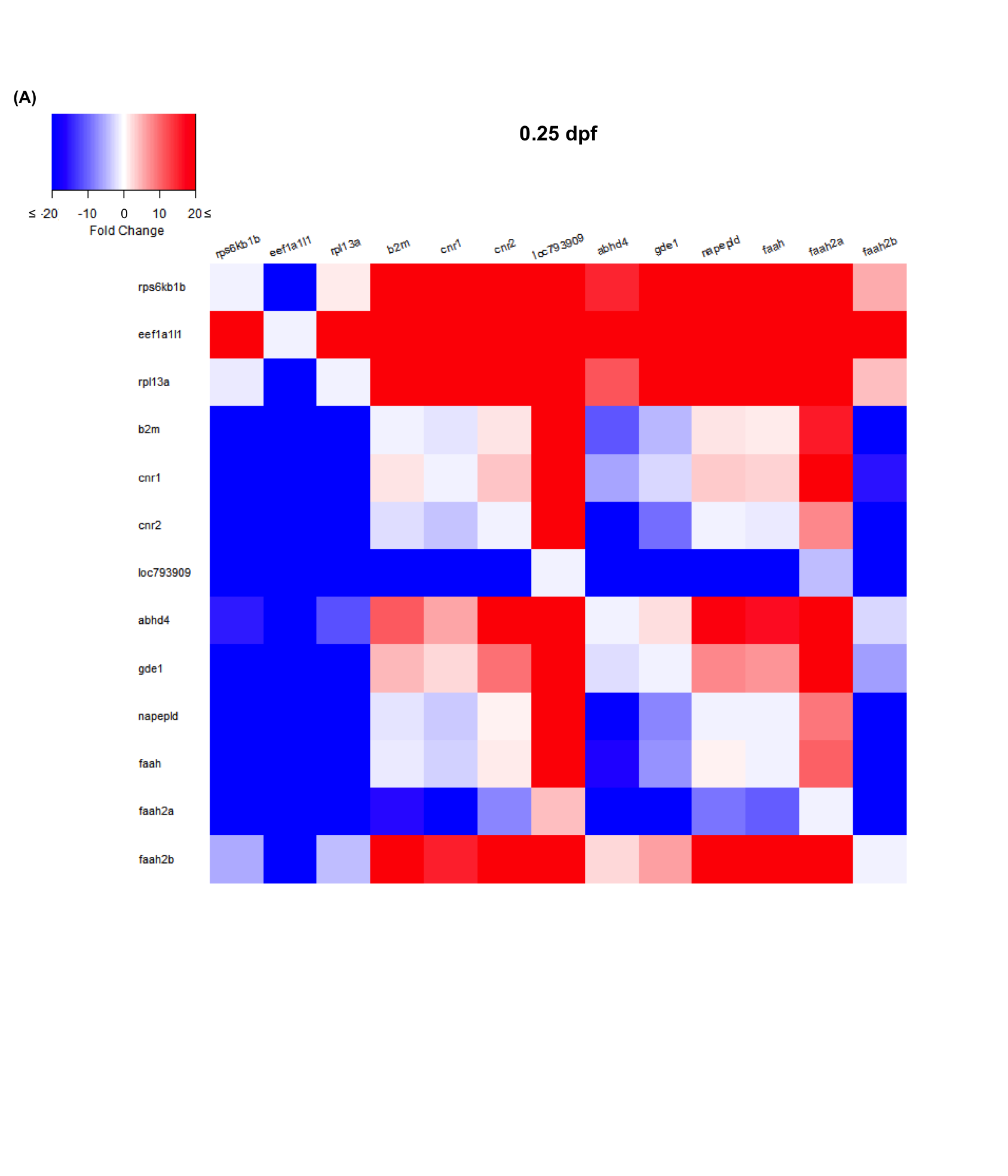

Supplement: S4 Fig — Fold change is calculated using the 2ΔCt formula when ΔCt is greater or equal to 0, and -2ΔCt when ΔCt is less than 0 for each gene pair combination. The coloring of the heatmaps is based on the calculation of the ΔCt by subtracting the Ct of the gene in the row from the Ct of the gene in the column (ΔCt = Ctcolumn−Ctrow). S4 Fig: 0.25 dpf, S5 Fig: 1 dpf, S6 Fig: 2 dpf, S7 Fig: 3 dpf, S8 Fig: 4 dpf, S9 Fig: 5 dpf, S10 Fig: 6 dpf, S11 Fig: 7 dpf. (TIF) [file pone.0190897.s004.tif]

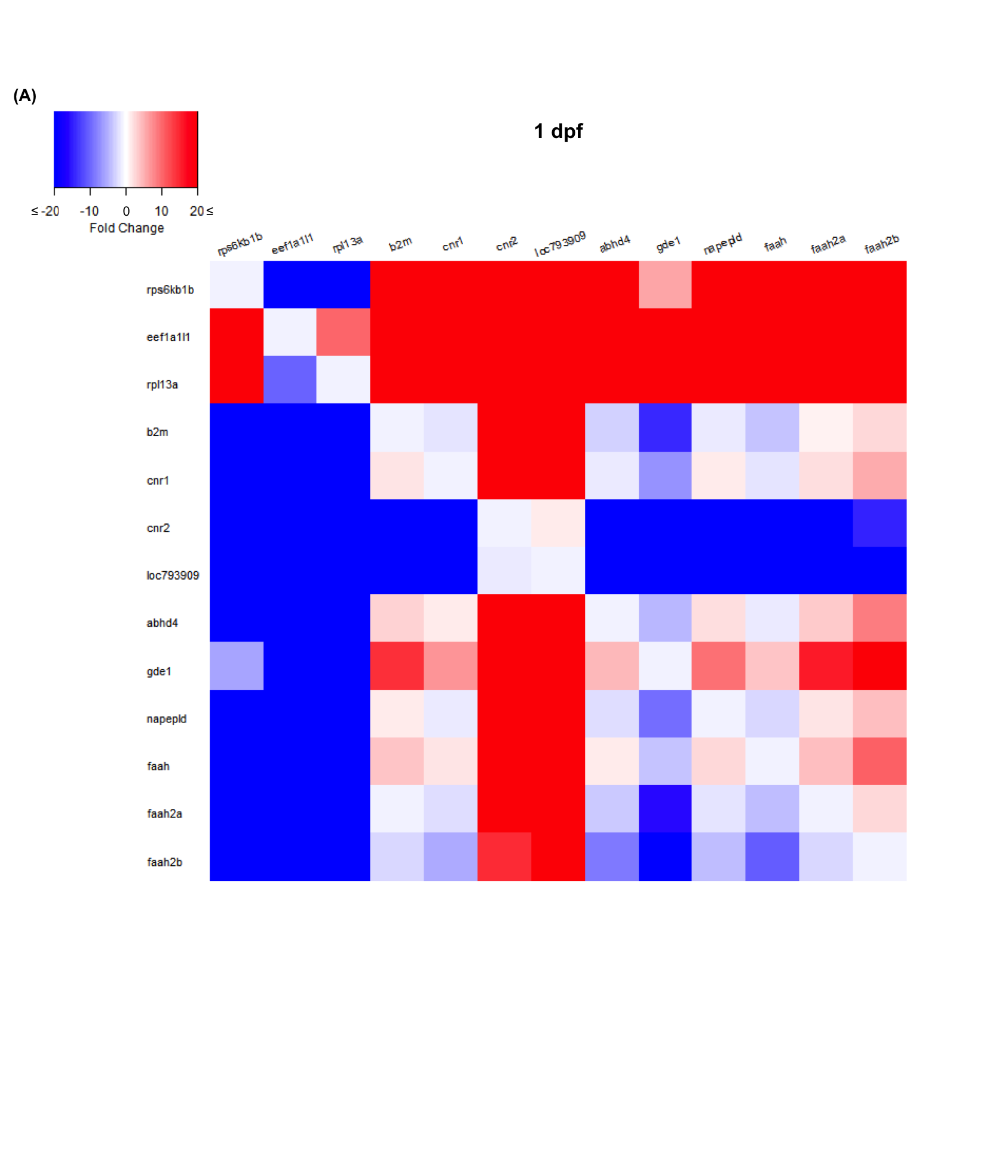

Supplement: S5 Fig — Fold change is calculated using the 2ΔCt formula when ΔCt is greater or equal to 0, and -2ΔCt when ΔCt is less than 0 for each gene pair combination. The coloring of the heatmaps is based on the calculation of the ΔCt by subtracting the Ct of the gene in the row from the Ct of the gene in the column (ΔCt = Ctcolumn−Ctrow). S4 Fig: 0.25 dpf, S5 Fig: 1 dpf, S6 Fig: 2 dpf, S7 Fig: 3 dpf, S8 Fig: 4 dpf, S9 Fig: 5 dpf, S10 Fig: 6 dpf, S11 Fig: 7 dpf. (TIF) [file pone.0190897.s005.tif]

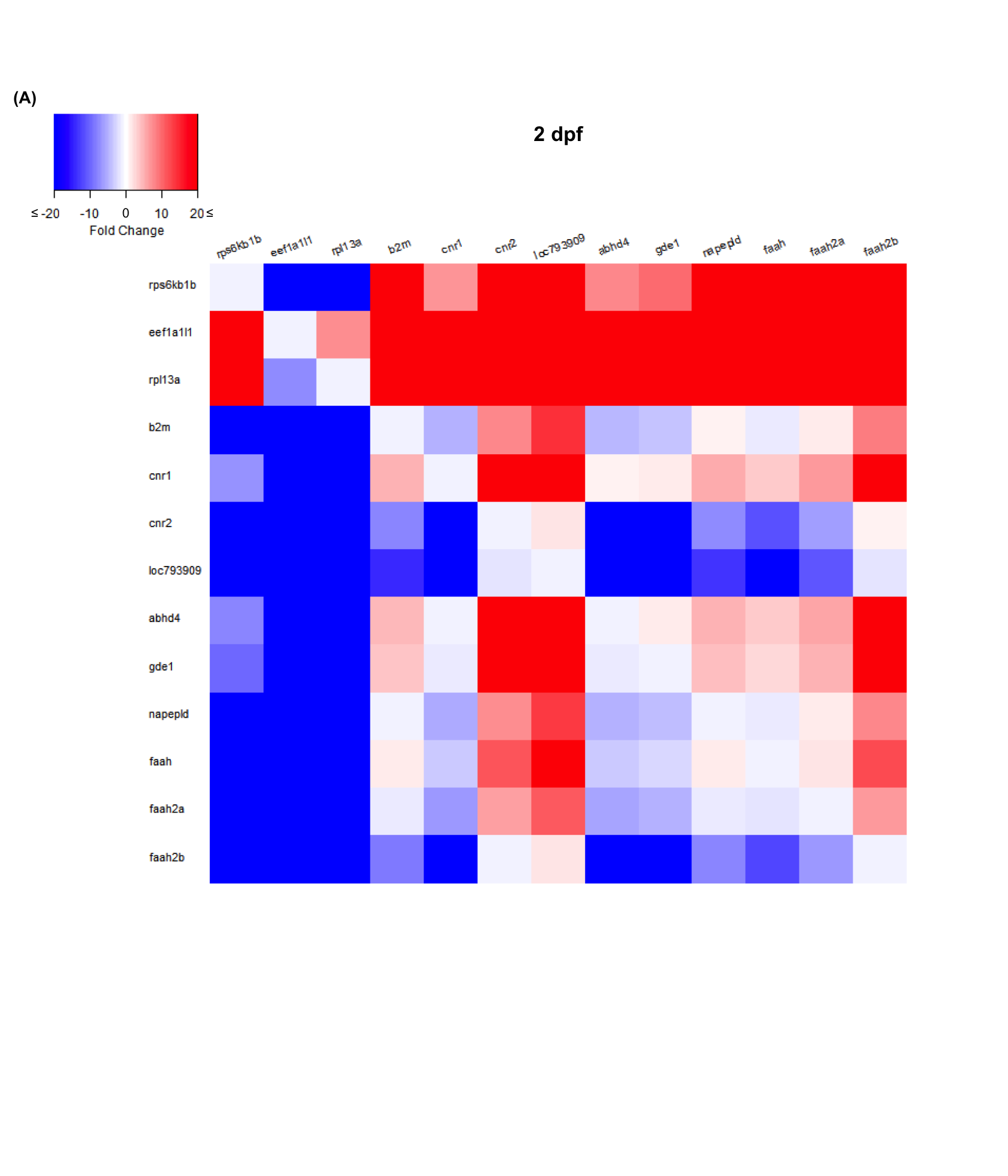

Supplement: S6 Fig — Fold change is calculated using the 2ΔCt formula when ΔCt is greater or equal to 0, and -2ΔCt when ΔCt is less than 0 for each gene pair combination. The coloring of the heatmaps is based on the calculation of the ΔCt by subtracting the Ct of the gene in the row from the Ct of the gene in the column (ΔCt = Ctcolumn−Ctrow). S4 Fig: 0.25 dpf, S5 Fig: 1 dpf, S6 Fig: 2 dpf, S7 Fig: 3 dpf, S8 Fig: 4 dpf, S9 Fig: 5 dpf, S10 Fig: 6 dpf, S11 Fig: 7 dpf. (TIF) [file pone.0190897.s006.tif]

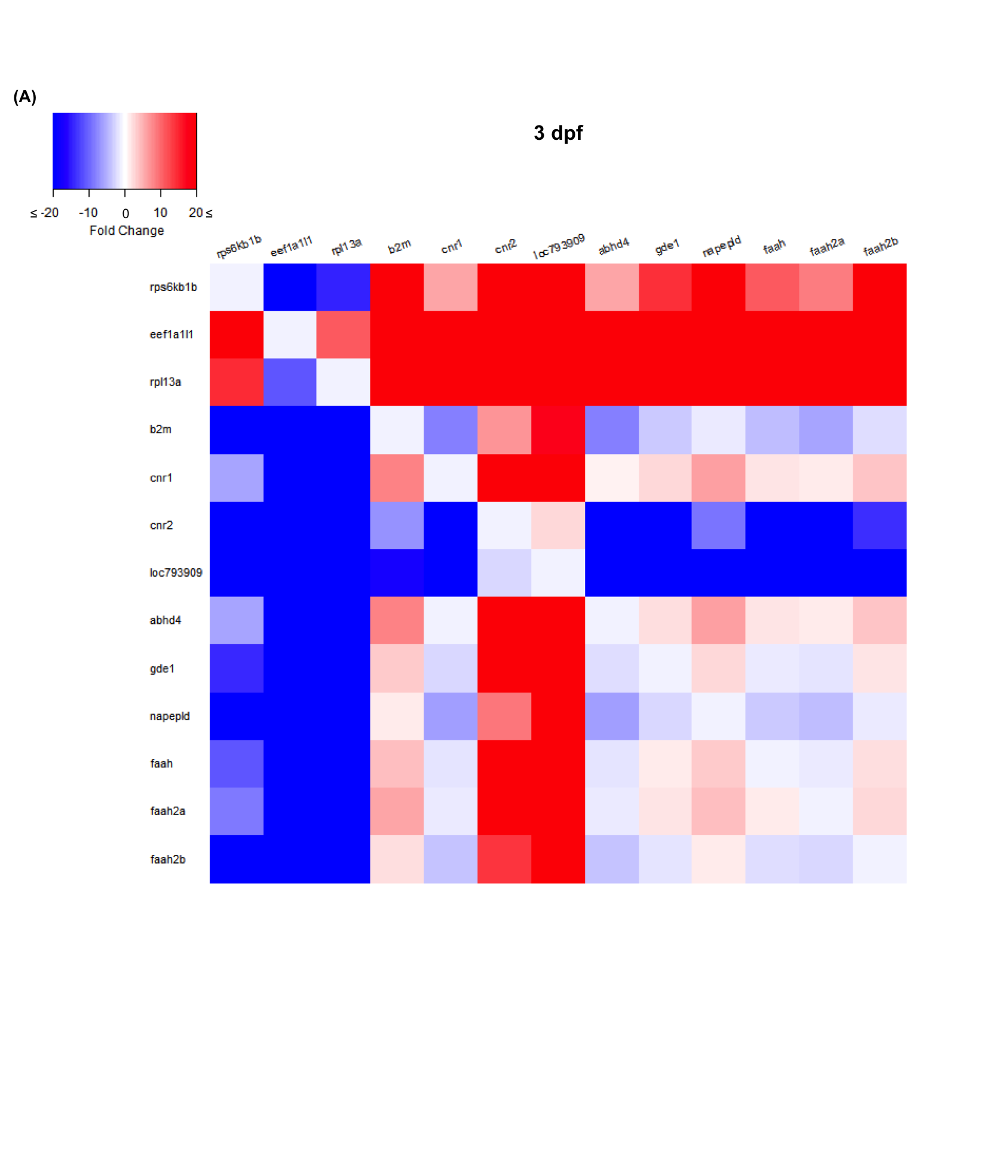

Supplement: S7 Fig — Fold change is calculated using the 2ΔCt formula when ΔCt is greater or equal to 0, and -2ΔCt when ΔCt is less than 0 for each gene pair combination. The coloring of the heatmaps is based on the calculation of the ΔCt by subtracting the Ct of the gene in the row from the Ct of the gene in the column (ΔCt = Ctcolumn−Ctrow). S4 Fig: 0.25 dpf, S5 Fig: 1 dpf, S6 Fig: 2 dpf, S7 Fig: 3 dpf, S8 Fig: 4 dpf, S9 Fig: 5 dpf, S10 Fig: 6 dpf, S11 Fig: 7 dpf. (TIF) [file pone.0190897.s007.tif]

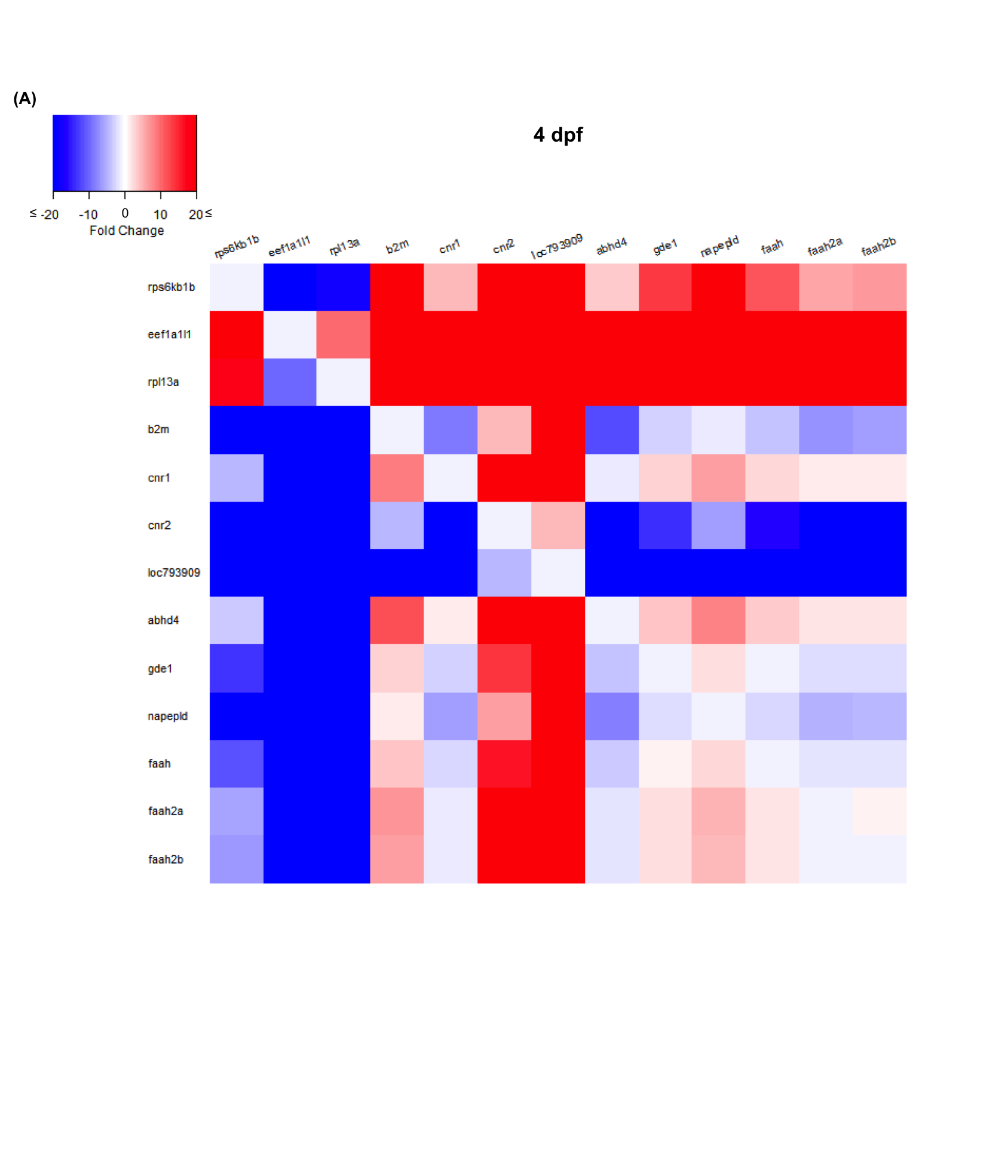

Supplement: S8 Fig — Fold change is calculated using the 2ΔCt formula when ΔCt is greater or equal to 0, and -2ΔCt when ΔCt is less than 0 for each gene pair combination. The coloring of the heatmaps is based on the calculation of the ΔCt by subtracting the Ct of the gene in the row from the Ct of the gene in the column (ΔCt = Ctcolumn−Ctrow). S4 Fig: 0.25 dpf, S5 Fig: 1 dpf, S6 Fig: 2 dpf, S7 Fig: 3 dpf, S8 Fig: 4 dpf, S9 Fig: 5 dpf, S10 Fig: 6 dpf, S11 Fig: 7 dpf. (TIF) [file pone.0190897.s008.tif]

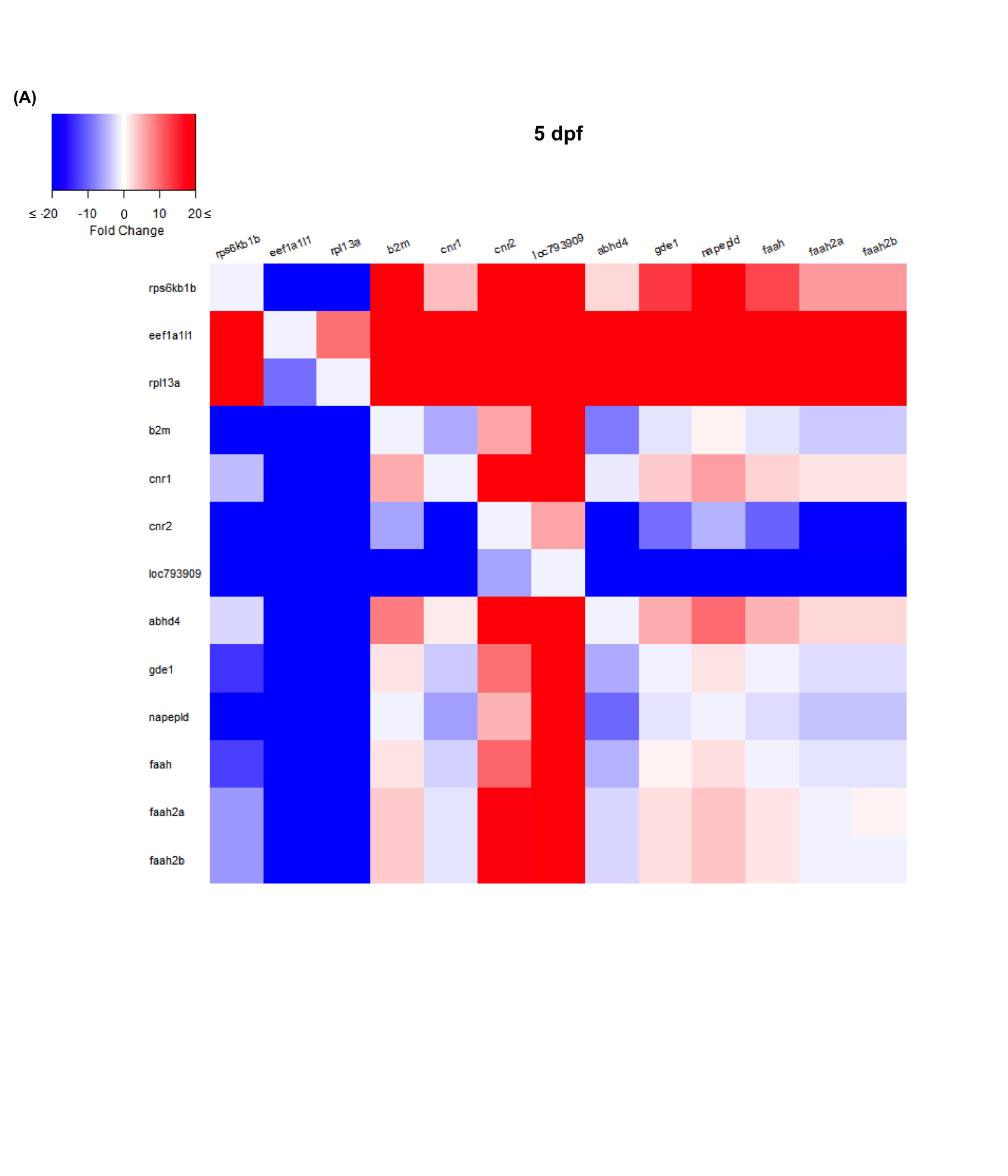

Supplement: S9 Fig — Fold change is calculated using the 2ΔCt formula when ΔCt is greater or equal to 0, and -2ΔCt when ΔCt is less than 0 for each gene pair combination. The coloring of the heatmaps is based on the calculation of the ΔCt by subtracting the Ct of the gene in the row from the Ct of the gene in the column (ΔCt = Ctcolumn−Ctrow). S4 Fig: 0.25 dpf, S5 Fig: 1 dpf, S6 Fig: 2 dpf, S7 Fig: 3 dpf, S8 Fig: 4 dpf, S9 Fig: 5 dpf, S10 Fig: 6 dpf, S11 Fig: 7 dpf. (TIF) [file pone.0190897.s009.tif]

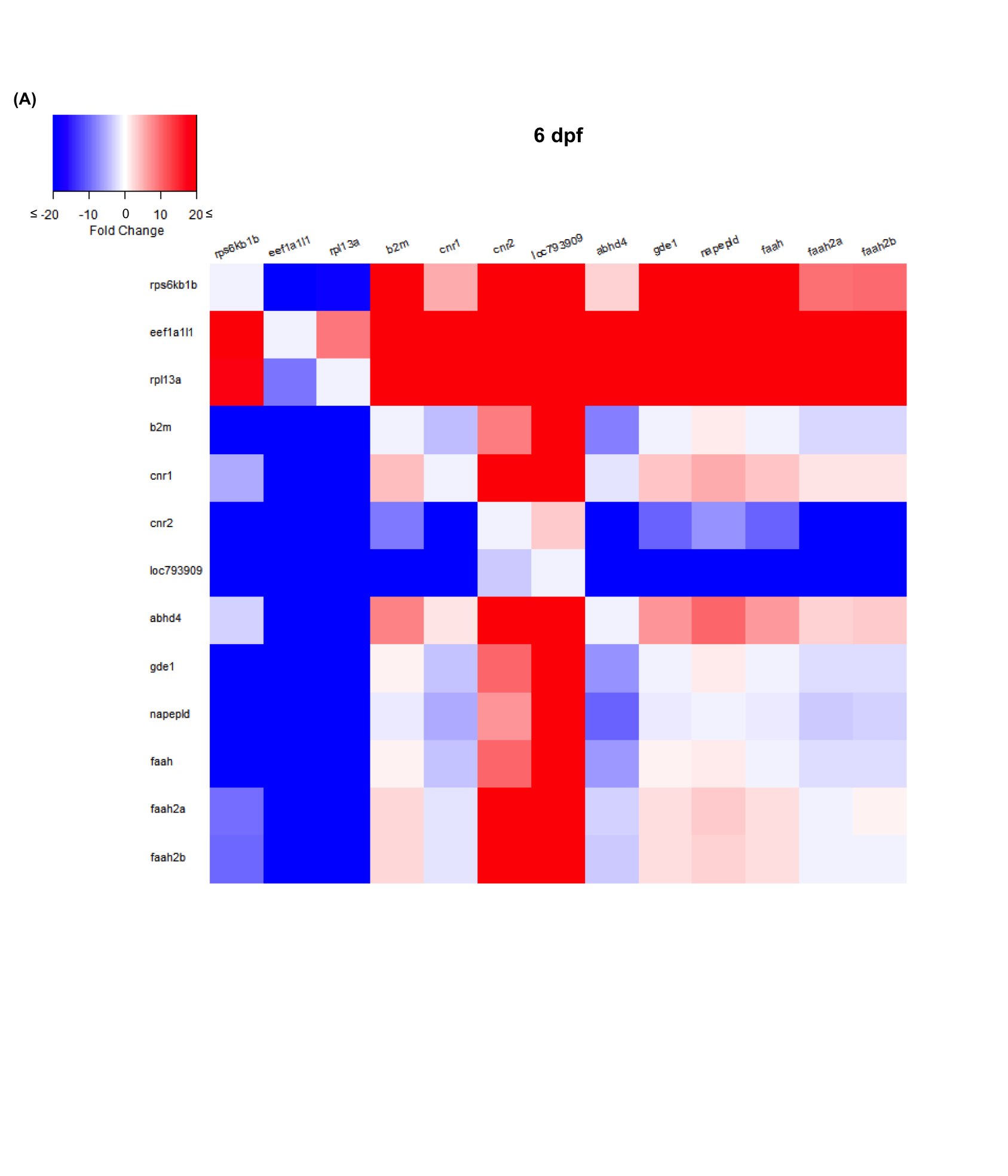

Supplement: S10 Fig — Fold change is calculated using the 2ΔCt formula when ΔCt is greater or equal to 0, and -2ΔCt when ΔCt is less than 0 for each gene pair combination. The coloring of the heatmaps is based on the calculation of the ΔCt by subtracting the Ct of the gene in the row from the Ct of the gene in the column (ΔCt = Ctcolumn−Ctrow). S4 Fig: 0.25 dpf, S5 Fig: 1 dpf, S6 Fig: 2 dpf, S7 Fig: 3 dpf, S8 Fig: 4 dpf, S9 Fig: 5 dpf, S10 Fig: 6 dpf, S11 Fig: 7 dpf. (TIF) [file pone.0190897.s010.tif]

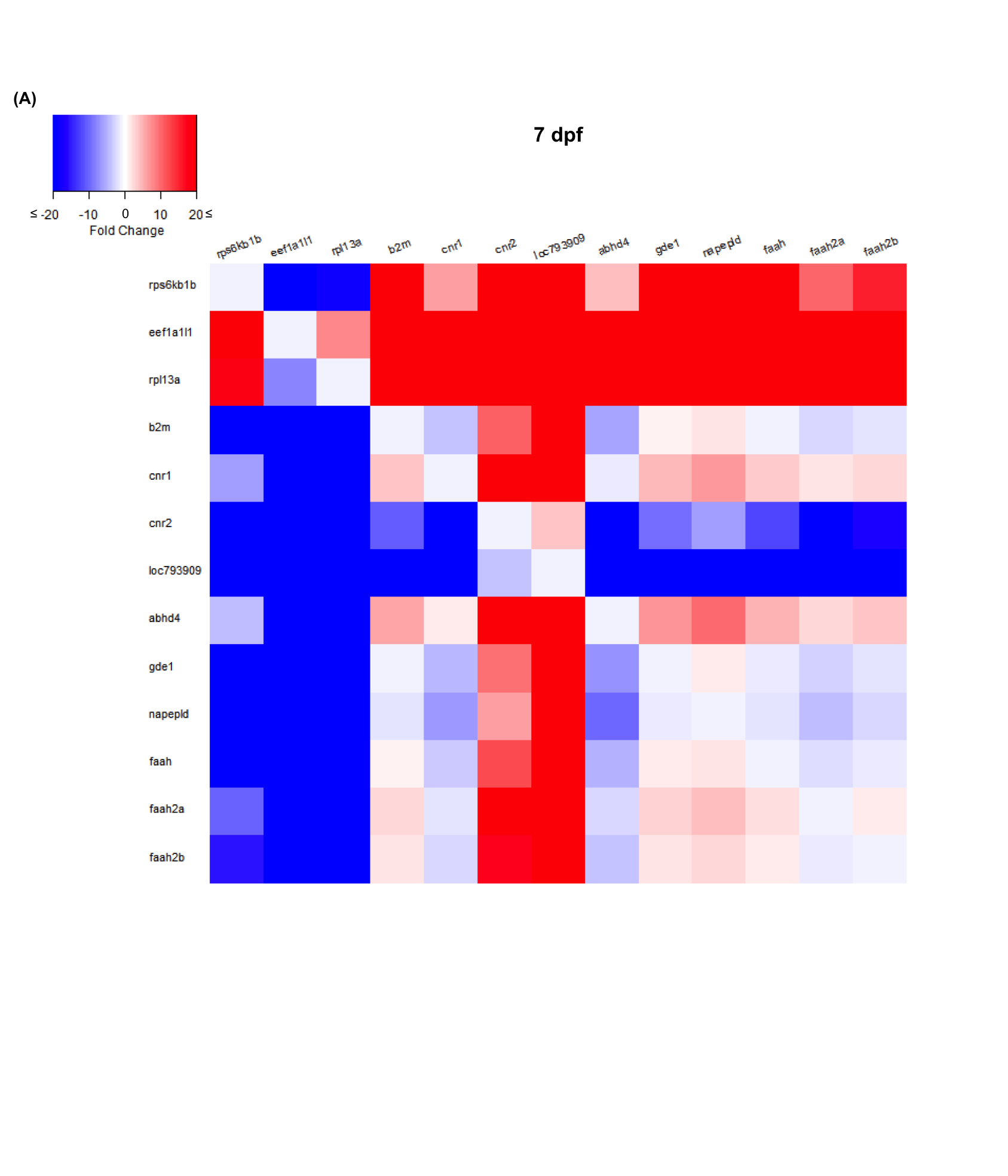

Supplement: S11 Fig — Fold change is calculated using the 2ΔCt formula when ΔCt is greater or equal to 0, and -2ΔCt when ΔCt is less than 0 for each gene pair combination. The coloring of the heatmaps is based on the calculation of the ΔCt by subtracting the Ct of the gene in the row from the Ct of the gene in the column (ΔCt = Ctcolumn−Ctrow). S4 Fig: 0.25 dpf, S5 Fig: 1 dpf, S6 Fig: 2 dpf, S7 Fig: 3 dpf, S8 Fig: 4 dpf, S9 Fig: 5 dpf, S10 Fig: 6 dpf, S11 Fig: 7 dpf. (TIF) [file pone.0190897.s011.tif]

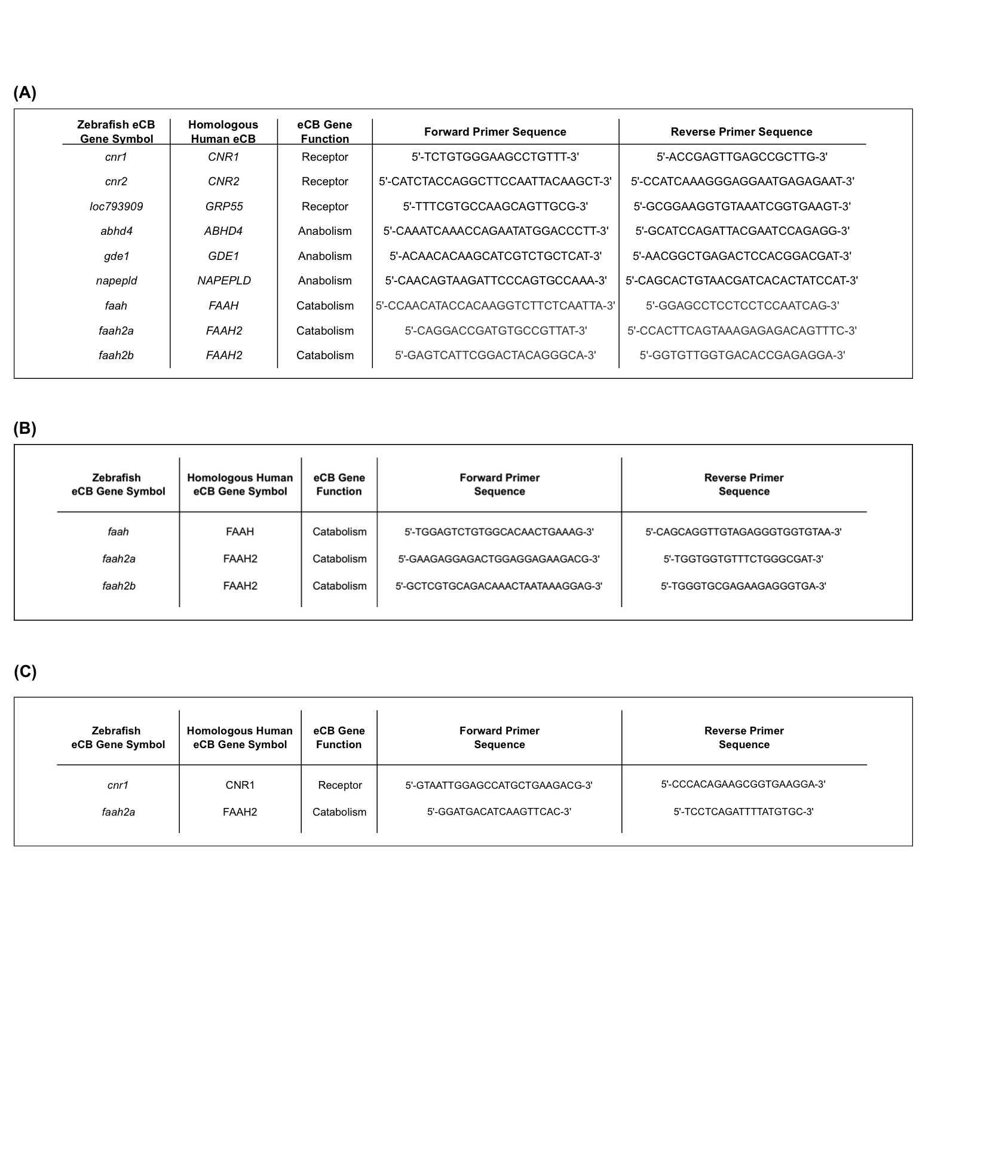

Supplement: S1 Table — (A) A list of select zebrafish eCB genes and the primers used to amplify target regions in them for qRT-PCR analyses. (B) A list of select zebrafish eCB genes and the primers used to amplify target regions in them for ISH analyses. (C) A list of select zebrafish eCB genes and the primers used to amplify target regions in them for RFLP analyses. (TIF) [file pone.0190897.s012.tif]

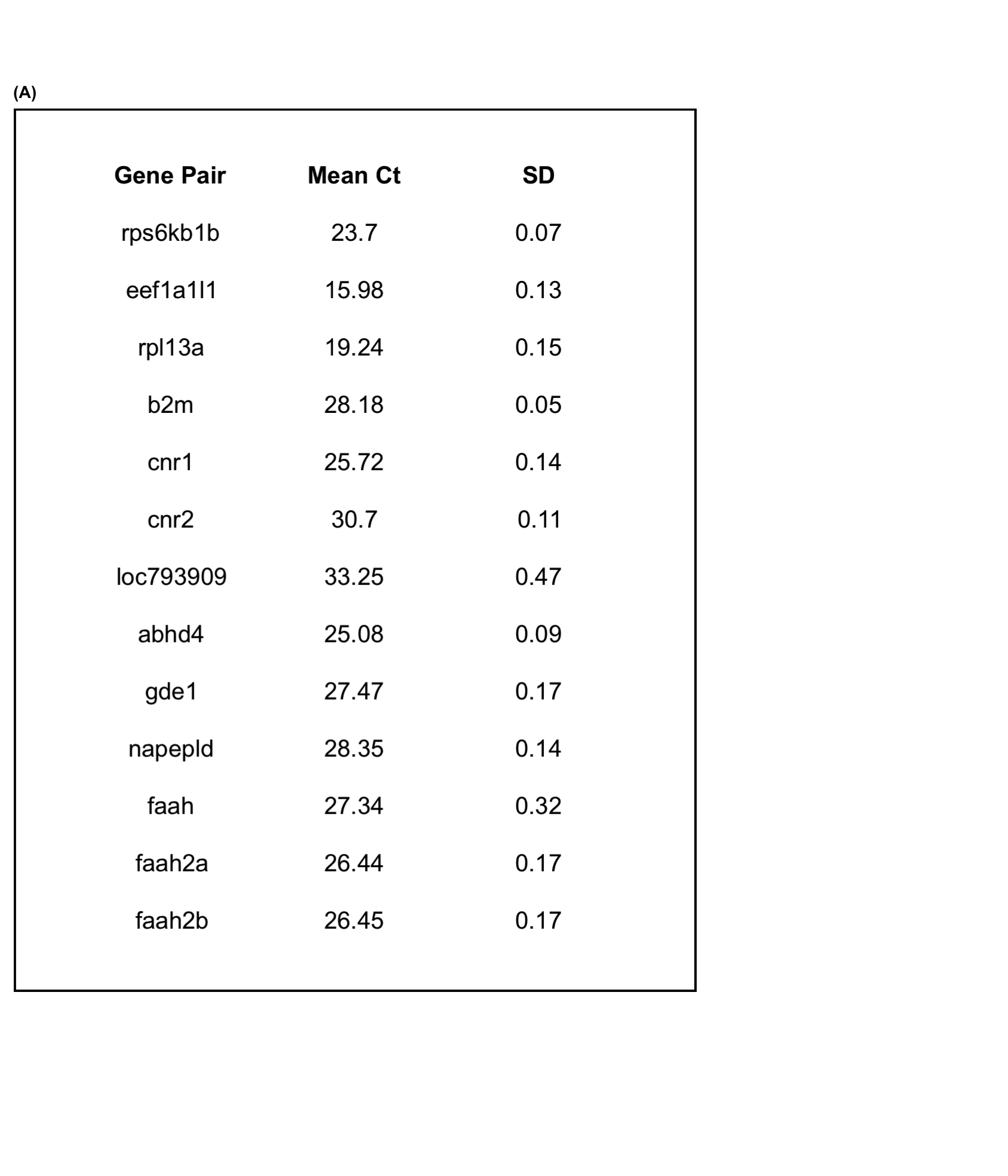

Supplement: S2 Table — (A) A list of Ct values for select zebrafish eCB and reference gene pairs at 5 dpf. The values are shown as a mean ± SD (10 larvae/n, n = 3). (TIF) [file pone.0190897.s013.tif]
